# Synthesis, fungicidal activity, structure-activity relationships (SARs) and density functional theory (DFT) studies of novel strobilurin analogues containing arylpyrazole rings

**DOI:** 10.1038/s41598-018-26154-5

**Published:** 2018-05-18

**Authors:** Yuanyuan Liu, Kunzhi Lv, Yi Li, Qiuli Nan, Jinyuan Xu

**Affiliations:** 1 Department of Chemical and Pharmaceutical Engineering, Southeast University ChengXian College, Nanjing, 210088 P. R. China; 2 Nanjing Sanhome Pharmaceutical Co Ltd, Nanjing, 210018 P. R. China; 3 College of Food Science and Light Industry, Nanjing Tech University, Nanjing, 211816 P. R. China

## Abstract

A series of novel strobilurin analogues (**1a-1f**, **2a-2e**, **3a-3e**) containing arylpyrazole rings were synthesized and characterized by NMR spectroscopy. The structures of **1f**, **2b** and **3b** were also determined by single crystal X-ray diffraction analysis. These analogues were collected together with other twenty-eight similar compounds **4a-4f**, **5a-5h**, **6a-6h** and **7a-7f** from our previous studies, for *in vitro* bioassays and thorough structure-activity relationships (SARs) studies. Most compounds exhibited excellent-to-good fungicidal activity against *Rhizoctonia solani*, especially **5c**, **7a**, **6c**, and **3b** with 98.94%, 83.40%, 71.40% and 65.87% inhibition rates at 0.1 μg mL^−1^, respectively, better than commercial pyraclostrobin. Comparative molecular field analysis (CoMFA) was employed to study three-dimensional quantitative structure-activity relationships (3D-QSARs). Density functional theory (DFT) calculation was also carried out to provide more information regarding SARs. The present work provided some hints for developing novel strobilurin fungicides.

## Introduction

**Figure 1:**
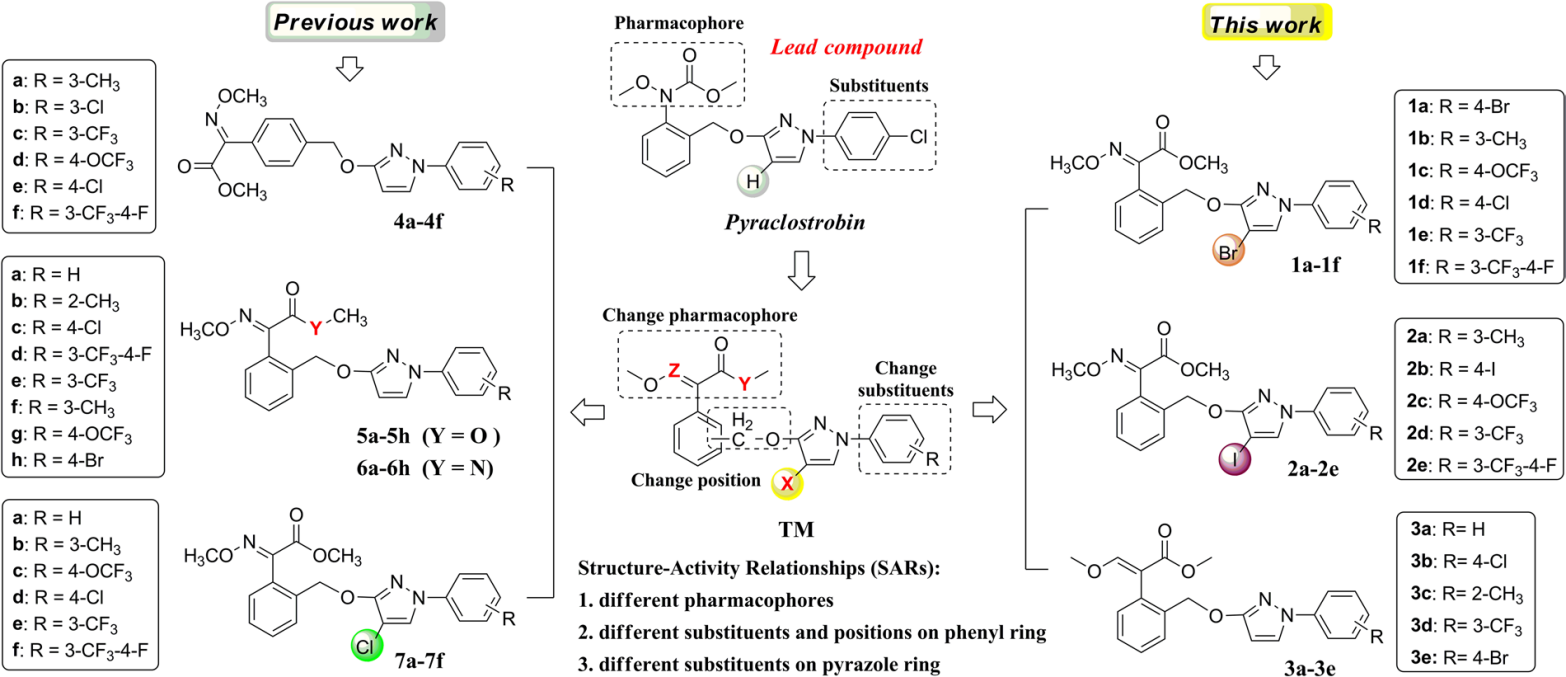
Design strategy of the target strobilurin analogues.

The resistance of pathogens has become one of the puzzling problems to crop protection, and the main solution is to develop novel fungicides with unique structures and mechanisms of action. Since the discovery of the strobilurin fungicide pyraclostrobin (Fig. 1), this novel fungicide class has occupied an important position due to its higher fungicidal activity, wider spectrum and lower toxicity toward mammalian cells^1–3^. Several representatives such as trifloxystrobin, kresoxim-methyl, metominostrobin and SYP-1620 have been commercialized (or marketed)^4,5^. Generally, the chemical structure of these strobilurins could be characterized by three parts: (*i*) a methyl (*E*)-*β*-methoxyiminoacetate or an isosteric methyl (*E*)-*β*-methoxyacrylate moiety as pharmacophore, *ii*) an aromatic bridge moiety, and (*iii*) a side chain. Combining the pharmacophore with a structurally diverse side chain is an effective way to get new strobilurin analogues, and the arylpyrazole structure of pyraclostrobin is such a side chain. In our previous work, the methoxyiminoacetate pharmacophore of trifloxystrobin was introduced into the arylpyrazole structure, and a series of strobilurin analogues **4a-4f**, **5a-5h**, **6a-6h** and **7a-7f** were synthesized (Fig. 1)^6–8^. However, their fungicidal activity and structure-activity relationships (SARs) have not been discussed together in detail.

As one of the most important methods in the design of new drugs with computer-aided drug design (CADD), three-dimensional quantitative structure-activity relationships (3D-QSARs) play an important role in the bioactivity prediction and structure optimization. The comparative molecular field analysis (CoMFA) proposed by Cramer *et al*.^9^ has become the most standard and universal method for 3D-QSAR study because of its good predictivity and intuitive image. For example, Li *et al*.^10^ reported a CoMFA 3D-QSAR model about a series of pyrazole derivatives against *Rhizoctonia solani*. Similarly Yang *et al*.^11^ reported fungicidal activity and 3D-QSAR study of phenylhydrazine substituted tetronic acid derivatives. Motivated by these findings, we conceived that establishing the 3D-QSAR model for our strobilurin analogues to predict the bioactivity and then optimize the structures might result in novel excellent fungicides.

According to the frontier-orbital theory, Highest Occupied Molecular Orbital (HOMO) and Lowest Unoccupied Molecular Orbital (LUMO) are the two significant factors that affect the bioactivities^12–14^. They establish the correlation in various chemical and biochemical systems. Recently, Li *et al*.^12,13^ and Zhu *et al*.^15,16^ have reported studies on the frontier-orbital energies of some novel active molecules, which provide useful information about the biological mechanism and for further structural optimization. Thus, the study of the frontier-orbital energies may be helpful to the investigation of fungicidal activity.

Taking all these into account, in this paper, in continuation of our studies on novel fungicidal strobilurin analogues, the methoxyiminoacetate pharmacophore of trifloxystrobin and methoxyacrylate pharmacophore of azoxystrobin were introduced into the halo-(un)substituted arylpyrazole structure, respectively, and a series of novel strobilurin analogues (**1a-1f**, **2a-2e**, **3a-3e**) were designed and synthesized (Fig. 1). The crystal structures of **1f**, **2b** and **3b** were verified, to stimulate a better understanding of their binding nature. The fungicidal activity of these analogues and other twenty-eight similar compounds **4a-4f**, **5a-5h**, **6a-6h** and **7a-7f** from our previous studies were investigated together, with the aim of thorough understanding the structure-activity relationships (SARs) and developing novel fungicides. Their 3D-QSAR model and density functional theory (DFT) studies were also carried out to provide some guidance for further structure modification.

## Results and Discussion

### Synthesis

**Figure 2:**
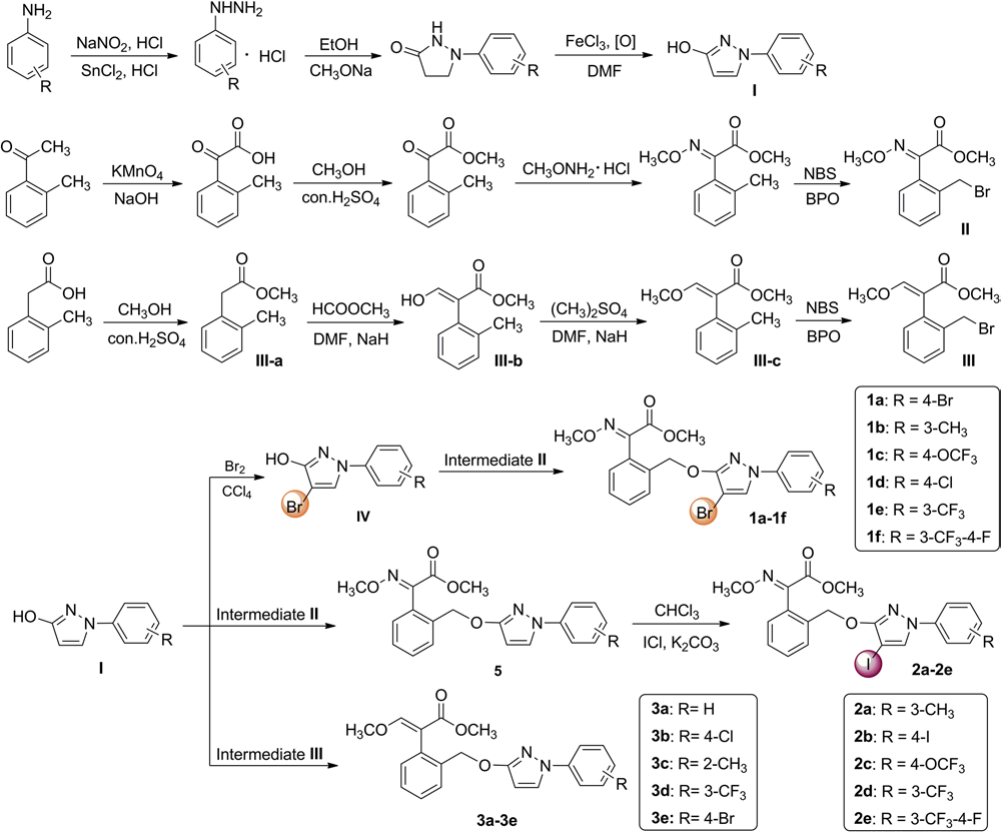
General synthetic routes of target products **1a-1f**, **2a-2e** and **3a-3e**.

**Figure 3:**
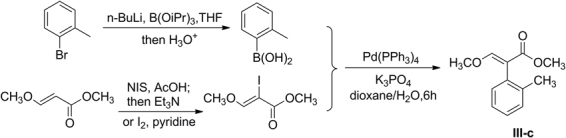
Suzuki-Miyaura coupling reaction for the synthesis of **III-c**.

General synthetic routes for final compounds **1a-1f**, **2a-2e** and **3a-3e** are shown in Fig. 2. Intermediates *N*-arylpyrazoles **I** were synthesized from arylhydrazines *via* addition-cyclization and oxidation, which could then afford 4-bromo-*N*-arylpyrazoles **IV** by bromination^17^. Intermediate benzyl bromide (*E*)-methyl 2-(2-(bromomethyl)phenyl)-2-(methoxyimino)acetate **II** was prepared from 1-(*o*-tolyl)ethanone *via* four steps including oxidation, esterification, oximation and bromination^8^. A previous report by Kim *et al*.^18^ described that intermediate (*E*)-methyl 3-methoxy-2-(*o*-tolyl)acrylate **III-c** could be synthesized from 1-bromo-2-methylbenzene and (*E*)-methyl 3-methoxyacrylate *via* Suzuki-Miyaura coupling reaction (Fig. 3). However, this approach required Grignard reagent and costly catalyst Pd(PPh_3_)_4_, which faced harsh reaction conditions and complicated processes. So in our procedure, readily accessible 2-(*o*-tolyl)acetic acid was used as starting material, and intermediate **III-c** could be obtained through three steps including esterification, condensation and methylation. The condensation of **III-a** with methyl formate was carried out under NaH alkaline condition, which gave **III-b** in 85% yield. A better yield (78%) of **III-c** was obtained in a molar ratio of **III-b** to dimethyl sulfate 1:1.2 equiv. in DMF as solvent, and with NaH as base.

**Figure 4:**
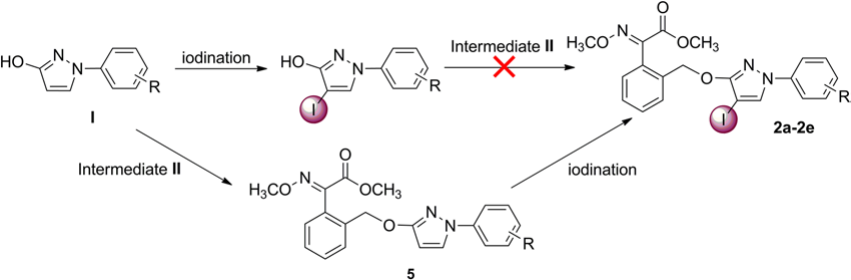
Synthesis of target products **2a-2e**.

In our previous studies, several strobilurin analogues (**4a-4f**, **5a-5h**, **6a-6h**, Fig. 1) have been prepared by the substitution of *N*-arylpyrazoles with benzyl bromide in acetone, using potassium carbonate (K_2_CO_3_) as acid-binding agent^7,8^. Motivated by this reaction, in our procedure, *N*-arylpyrazoles **IV** and **I** were allowed to react with benzyl bromide **II** and **III**, respectively, in a molar 1:1.1 equiv. in boiling acetone in the presence of K_2_CO_3_, which afforded the target (*E*)-methyl 2-(2-(((4-bromo-1-aryl-1*H*-pyrazol-3-yl)oxy)methyl)phenyl)-2-(methoxyimino)acetate (**1a-1f**) and (*E*)-methyl 3-methoxy-2-(2-(((1-aryl-1*H*-pyrazol-3-yl)oxy)methyl)phenyl)acrylate (**3a-3e**) in 78–85% and 72–78% yields, respectively, as sole isolable products (Fig. 2). However, the target products (*E*)-methyl 2-(2-(((4-iodo-1-aryl-1*H*-pyrazol-3-yl)oxy)methyl)phenyl)-2-(methoxyimino)acetate (**2a-2e**) could not be obtained by the similar methods like **1a-1f** *via* iodination first and then substitution (Fig. 4). Because the iodine was a better leaving group which made the bond ruptures more easily when the substitution took place. Therefore, compounds **5** were proposed to be synthesized firstly, which then underwent iodination to give products **2a-2e** in 80–88% isolated yields with good functional-group tolerance. The iodination was carried out in a molar ratio of **5** to iodine monochloride (ICl) 1:2 equiv. in CHCl_3_ as solvent, and with K_2_CO_3_ as acid-binding agent. Other iodine reagents such as I_2_, *N*-iodosuccinimide (NIS), KI and NaI were also selected. However, the results were unsatisfactory. The regioselectivity of the reactions and the structures of the products **1a-1f**, **2a-2e** and **3a-3e** were unequivocally determined by NMR spectroscopy and single-crystal X-ray diffraction analysis of (*E*)-methyl 2-(2-(((4-bromo-1-(4-fluoro-3-(trifluoromethyl)phenyl)-1*H*-pyrazol-3-yl)oxy)methyl)phenyl)-2-(methoxyimino)acetate (**1f**), (*E*)-methyl 2-(2-(((4-iodo-1-(4-iodophenyl)-1*H*-pyrazol-3-yl)oxy)methyl)phenyl)-2-(methoxyimino)acetate (**2b**) and (*E*)-methyl 2-(2-(((1-(4-chlorophenyl)-1*H*-pyrazol-3-yl)oxy)methyl)phenyl)-3-methoxyacrylate (**3b**).

### Structure

The structures of **1a-1f**, **2a-2e** and **3a-3e** were confirmed by their NMR spectra. In the ^1^H NMR spectra, as a result of the deshielding effect of bromo and iodo groups, the CH of the pyrazole ring in **1a-1f** and **2a-2e** appeared as a singlet at low field *δ* 7.56–7.74 ppm and *δ* 7.65–7.81 ppm, respectively, whereas the two CH protons in **3a-3e** appeared at *δ* 7.54–7.80 ppm and *δ* 5.73–5.83 ppm, respectively, as two doublets with coupling constants around 2.5 Hz. The CH_2_ in the oxy side chain appeared as a singlet at *δ* 5.08–5.26 ppm. The aromatic protons of all products resonated in the range of *δ* 7–8 ppm in ^1^H NMR spectra, and the ^13^C NMR signals were observed around *δ* 110–140 ppm. The chemical shifts of methoxy (OCH_3_) H-atoms appeared as two singlets around *δ* 3.67–4.09 ppm and *δ* 3.57–3.90 ppm, respectively. All products exhibited a carbonyl (C=O) ^13^C signal at the lowest field in the region of *δ* 162.2–168.1 ppm.

**Figure 5:**
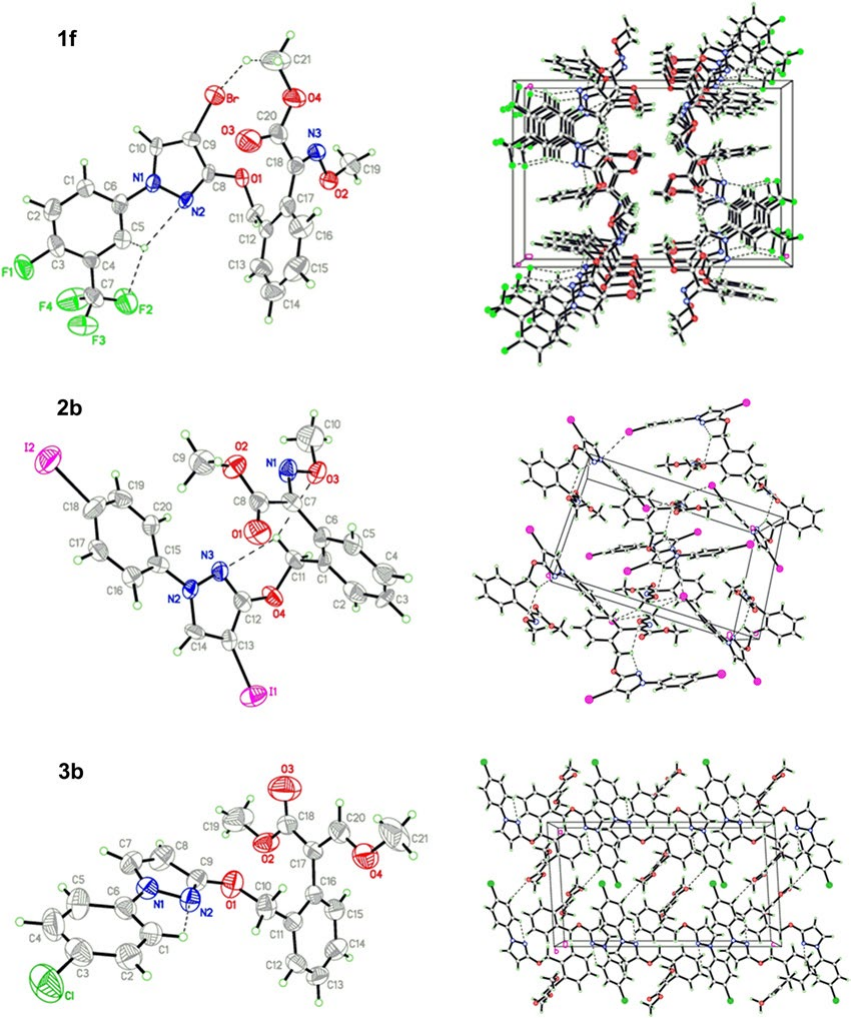
X-ray crystal structures and packing diagrams of **1f**, **2b** and **3b**.

The detailed crystal and structure refinement data of products **1f**, **2b** and **3b** are listed in Table 1. Both **1f** and **3b** crystallize in a monoclinic space group *P*2_1_/c, whereas **2b** crystallizes in a monoclinic space group *P*2_1_/n. In the crystal structures (Fig. 5), the bond lengths of C13-I1 (2.046(11) Å) and C18-I2 (2.070(12) Å) in **2b** are longer than C9-Br (1.851(6) Å) in **1f**, which represent a typical C-I bond length, and the values are similar to those (2.071(3) and 2.070(4)) reported for other related derivatives^19^. The bond lengths of C8-O1 (1.312(7) Å) in **1f**, C12-O4 (1.341(12) Å) in **2b** and C9-O1 (1.337(5) Å) in **3b** are longer than standard C=O double bond (1.229 Å), and belong to C-O single bond. The bond angle of C8-C9-C10 in **1f** is 105.2(6)°, whereas the corresponding bond angles in **2b** and **3b** are 105.3(9)° (C12-C13-C14) and 104.3(4)° (C7-C8-C9), respectively. These values are similar to the typical angles of five-membered ring (108.0°). The bridge benzene ring *A* and the terminal benzene ring *B* are connected to the pyrazole ring *C*, twisted by 81.58° and 15.00° (**1f**), 59.58° and 21.11° (**2b**), 58.01° and 9.99° (**3b**), respectively, whereas they form a dihedral angle of 88.74°, 58.98° and 64.63°, respectively. The ester and methoxyimino (**1f** and **2b**) or methoxyethene (**3b**) are almost coplanar, twisted by 76.21° and 68.47° (*A* in **1f**), 59.43° and 2.72° (*A* in **2b**), 80.78° and 89.15° (*A* in **3b**), 14.52° and 23.67° (*B* in **1f**), 21.51° and 61.79° (*B* in **2b**), 80.84° and 78.88° (*B* in **3b**), 22.58° and 29.97° (*C* in **1f**), 8.82° and 22.45° (*C* in **2b**), 84.90° and 86.31° (*C* in **3b**), respectively, from the planes of the rings *A*, *B* and *C*. The intramolecular C5-H5A···F2 and C5-H5A···N2 H-bonds in **1f**, C11-H11A···N3 H-bond in **2b**, and C1-H1A···N2 H-bond in **3b** result in the formation of four non-planar pseudo-rings *D* (C5/C4/C7/F2/H5A), *E* (N1/N2/H5A/C5/C6), *F* (N3/C12/O4/C11/H11A) and *G* (N1/N2/H1A/C1/C6). In the crystal, six intermolecular H-bonds in **1f** (C1-H1A···O3, C10-H10A···O3, C14-H14A···F1, C14-H14A···F4, C16-H16A···N2 and C21-H21B···Br), two intermolecular H-bonds in **2b** (C14-H14A···O1 and C16-H16A···O1) and one intermolecular H-bond in **3b** (C2-H2B···O3) reinforce the crystal packing (Fig. 5). These crystallographic data could provide a basis for elucidating the effect on their biological activities.

**Table 1:** Crystallographic data and structure refinement for compounds **1f**, **2b** and **3b**.

	**1f**	2b	3b
Empirical formula	C_21_H_15_BrF_4_N_3_O_4_	C_20_H_17_I_2_N_3_O_4_	C_21_H_19_ClN_2_O_4_
CCDC number	1433534	1433532	1433533
Formula weight	529.27	617.17	398.83
Temperature [K]	293(2)	293(2)	293(2)
Wavelength [Å]	0.71073	0.71073	0.71073
Crystal system	Monoclinic	Monoclinic	Monoclinic
Space group	P21/c	P21/n	P21/c
Unit cell dimensions			
*a* [Å]	20.446 (4)	11.639 (2)	11.773(2)
*b* [Å]	13.551(3)	16.736(3)	7.8560(16)
*c* [Å]	7.0590(14)	12.343(3)	21.480(4)
*α* [°]	90.00	90.00	90.00
*β* [°]	90.10(3)	116.01(3)	93.27(3)
*γ* [°]	90.00	90.00	90.00
Volume [Å^3^]	1955.8(7)	2160.8(7)	1983.4(7)
*Z*	4	4	4
*ρ*_calcd_ [g cm^−3^]	1.797	1.897	1.336
*μ* [mm^−1^]	2.177	2.941	0.222
*F*(000)	1060	1184	832
Crystal size [mm^3^]	0.20 × 0.10 × 0.10	0.20 × 0.10 × 0.10	0.30 × 0.20 × 0.10
*θ* range [°] for data collection	1.99 to 28.18	2.01 to 25.42	1.73 to 25.36
Index ranges	−24 ≤ h ≤ 0	0 ≤ h ≤ 14	0 ≤ h ≤ 14
	−16 ≤ k ≤ 0	0 ≤ k ≤ 20	0 ≤ k ≤ 9
	−9 ≤ l ≤ 9	−14 ≤ l ≤ 13	−25 ≤ l ≤ 25
Reflections collected	4077	4139	3824
Independent reflections	3967 [*R*_int_ = 0.0997]	3941 [*R*_int_ = 0.0990]	3637 [*R*_int_ = 0.0657]
Max. and min. transmission	0.8117/0.6699	0.7574/0.5908	0.9781/0.9364
Data/restraints/parameters	3967/2/298	3941/0/262	3637/1/253
Goodness-of-fit on *F*^2^	1.004	1.009	1.008
Final *R* indices [*I* > 2*σ*(*I*)]; *R*_1_, *wR*_2_	0.0772, 0.1344	0.0791, 0.1683	0.0771, 0.1690
*R*_1_, *wR*_2_ (all data)	0.1942, 0.1657	0.1544, 0.1963	0.1452, 0.1997
Largest diff. peak and hole [e·Å^−3^]	0.383 and −0.404	0.522 and −0.305	1.051 and −0.309

### Fungicidal Activity and Structure-Activity Relationships (SARs)

**Figure 6:**
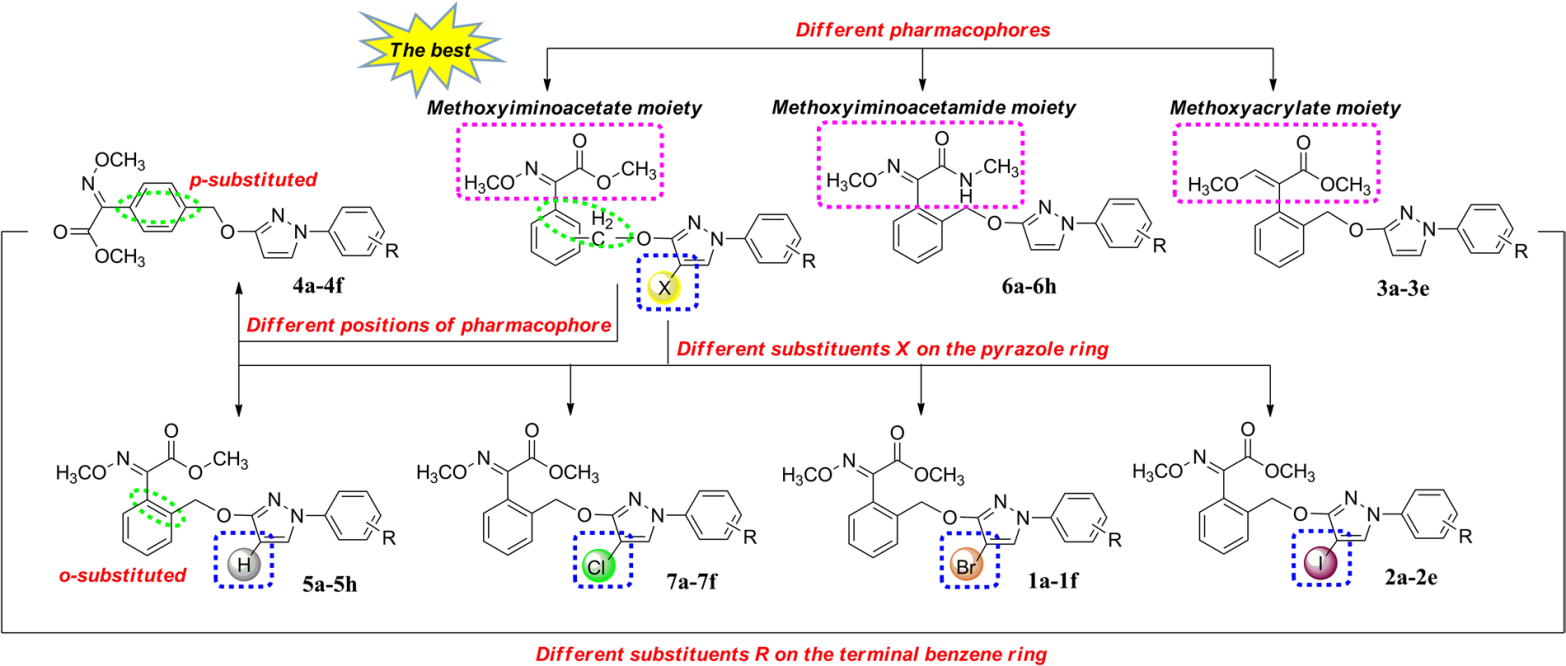
The structure-activity relationships (SARs) of forty-four strobilurin analogues.

As an important method of drug molecular design, the structure-activity relationships (SARs) can provide the guidance and enlightenment to the bioactivity prediction and structure optimization. In our previous studies, twenty-eight compounds **4a-4f**, **5a-5h**, **6a-6h** and **7a-7f** with similar strobilurin pharmacophores have been synthesized (Fig. 1). However, their SARs have not been discussed together. Here, we wish to report the SARs of all these forty-four strobilurin analogues (including sixteen compounds **1a-1f**, **2a-2e** and **3a-3e** synthesized in this paper) from the following three aspects: (*i*) the effect of different pharmacophores and their positions; (*ii*) the effect of different substituents R on the terminal benzene ring; (*iii*) the effect of different substituents X on the pyrazole ring (Fig. 6).

**Figure 7:**
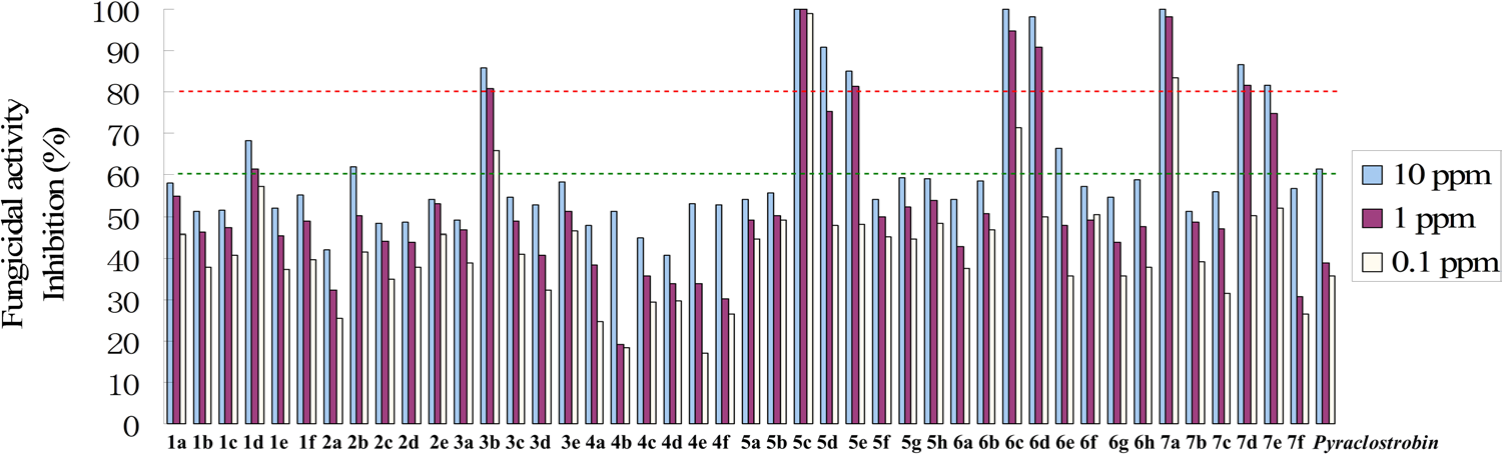
The fungicidal activity of forty-four strobilurin analogues against *Rhizoctonia solani*.

Compounds **1a-1f**, **2a-2e**, **3a-3e**, **4a-4f**, **5a-5h**, **6a-6h** and **7a-7f** were screened for bioactivity against two fungi, namely *Rhizoctonia cerealis* and *Gibberella zeae*, at the dosages of 10 μg mL^−1^, 1 μg mL^−1^ and 0.1 μg mL^−1^, respectively. As can be seen in Table 2 and Fig. 7, most compounds have excellent-to-good fungicidal activity against *Rhizoctonia solani* at 10 μg mL^−1^, especially **5c**, **6c** and **7a** with 100% antifungal activity, as well as **3b**, **5d**, **5e**, **6d**, **7d** and **7e** with more than 80% antifungal activity. When the concentration was reduced to 0.1 μg mL^−1^, **5c**, **7a**, **6c**, and **3b** also had 98.94%, 83.40%, 71.40% and 65.87% inhibition rates, which were much better than commercial pyraclostrobin. This might imply that the introduction of suitable pharmacophores by taking the electronic effect and substituted positions into full consideration was important for improving the fungicidal activity. However, most compounds showed weak fungicidal activity against *Gibberella zeae* except **5c**, **6b**, **6c**, **6e** and **7d** with moderate inhibitory activity at 10 μg mL^−1^. Therefore, the SARs based on the fungicidal activity against *Rhizoctonia solani* were discussed as follows.

**Table 2:** Fungicidal activity of forty-four strobilurin analogues.

Structure	No.	Inhibition [%]^[a]^					
		*Rhizoctonia solani*			*Gibberella zeae*		
		10	1	0.1	10	1	0.1
	**1a**	57.97	54.73	45.68	30.28	12.31	0.32
	**1b**	51.10	46.27	37.89	5.32	0.00	0.00
	**1c**	51.33	47.28	40.69	12.69	9.17	0.00
	**1d**	68.18	61.51	57.10	35.32	21.41	12.56
	**1e**	51.85	45.32	37.24	17.45	7.52	0.32
	**1f**	55.21	48.76	39.53	18.25	0.00	0.00
	**2a**	42.02	32.18	25.37	4.79	0.00	0.00
	**2b**	62.02	50.07	41.57	36.04	0.00	0.00
	**2c**	48.21	44.22	34.93	0.00	0.00	0.00
	**2d**	48.47	43.96	37.85	9.58	3.96	0.00
	**2e**	54.05	52.99	45.55	13.96	10.00	8.54
	**3a**	49.18	46.65	38.73	33.23	4.52	0.32
	**3b**	**85.78**	**80.86**	**65.87**	48.56	19.49	10.54
	**3c**	54.72	48.73	40.82	21.98	8.74	0.32
	**3d**	52.83	40.57	32.41	22.15	9.87	0.18
	**3e**	58.21	51.13	46.56	19.49	5.31	0.28
	**4a**	47.73	38.31	24.67	10.34	8.91	8.62
	**4b**	51.27	19.14	18.43	19.83	15.23	8.62
	**4c**	44.85	35.68	29.38	23.85	10.63	1.44
	**4d**	40.68	33.97	29.73	10.63	6.03	0.57
	**4e**	53.04	33.97	17.02	15.80	9.20	8.33
	**4f**	52.68	30.08	26.55	25.57	8.62	0.57
	**5a**	54.02	48.98	44.51	37.65	28.34	18.85
	**5b**	55.58	50.22	49.15	35.34	27.30	17.82
	**5c**	**100.00**	**100.00**	**98.94**	62.53	51.32	34.77
	**5d**	**90.82**	**75.28**	**47.74**	40.23	47.41	29.02
	**5e**	**85.17**	**81.29**	**48.09**	36.49	34.20	33.33
	**5f**	54.05	49.80	45.02	36.56	14.38	0.62
	**5g**	59.36	52.19	44.49	42.50	31.04	0.00
	**5h**	59.10	53.78	48.21	38.47	23.04	15.56
	**6a**	53.97	42.84	37.56	48.34	39.31	17.72
	**6b**	58.47	50.53	46.68	50.86	35.92	16.95
	**6c**	**100.00**	**94.70**	**71.40**	56.15	44.48	33.62
	**6d**	**98.23**	**90.82**	**49.86**	46.55	35.63	31.03
	**6e**	66.53	47.68	35.72	50.63	48.54	0.00
	**6f**	57.24	49.00	50.33	42.92	21.88	9.58
	**6g**	54.65	43.72	35.57	46.25	36.25	5.00
	**6h**	58.84	47.41	37.87	36.88	26.04	0.00
	**7a**	**100.00**	**98.23**	**83.40**	39.37	32.76	26.15
	**7b**	51.05	48.57	39.04	37.36	34.48	25.00
	**7c**	55.86	47.03	31.50	34.48	33.91	31.61
	**7d**	**86.58**	**81.64**	**50.21**	51.08	37.61	34.89
	**7e**	**81.64**	**74.93**	**51.98**	33.33	33.05	33.05
	**7f**	56.57	30.79	26.55	30.17	5.17	4.60
		61.51	38.91	35.73	35.06	6.32	0.57

^a^0, No activity, and 100, total kill.

In terms of the *o*-substituted pharmacophores, the sequence of fungicidal activity against *Rhizoctonia solani* was methoxyiminoacetate moiety (**5a-5h**) > methoxyiminoacetamide moiety (**6a-6h**) > methoxyacrylate moiety (**3a-3e**) in general, irrespective of difference in substituent R on the terminal phenyl ring. For example, within the series of R = 4-Cl derivatives, methoxyiminoacetate-derivative **5c** displayed a much higher fungicidal activity than the corresponding methoxyiminoacetamide-derivative **6c**, while the methoxyacrylate-derivative **3b** showed the lowest. Similar speculation could apply to the compounds **5e**, **6e** and **3d** (R = 3-CF_3_). All the above three chloro-containing compounds **5c**, **6c** and **3b** showed better activity than pyraclostrobin, which indicated the methoxycarbamate pharmacophore of pyraclostrobin might have no effective impact on the inhibition of *Rhizoctonia solani*. In addition, when changing the best *o*-substituted methoxyiminoacetate pharmacophore into the *p*-substituted, the results were unsatisfactory. Compounds **4a-4e** showed much lower fungicidal activity than **5a-5h**, which indicated the significant impact of pharmacophore position on the inhibition rates, and the *o*-substitution might be better.

To examine the electronic effect of substituent R on the phenyl ring, the electron-donating CH_3_ and electron-withdrawing F, Cl, Br, I, CF_3_ were introduced. Compounds with electron-withdrawing substituents displayed higher fungicidal activity against *Rhizoctonia solani* than that with electron-donating substituents, as seen in the comparison of **1d** (R = 4-Cl) *vs*. **1b** (R = 3-CH_3_), **2b** (R = 4-I) *vs*. **2a** (R = 3-CH_3_), **3b** (R = 4-Cl) *vs*. **3c** (R = 2-CH_3_), **5c** (R = 4-Cl) *vs*. **5b** (R = 2-CH_3_), **6c** (R = 4-Cl) *vs*. **6b** (R = 2-CH_3_), and **7d** (R = 4-Cl) *vs*. **7b** (R = 3-CH_3_). According to the different electronic effect of electron-withdrawing substituent R, the sequence of fungicidal activity against *Rhizoctonia solani* is chloro-substituted > bromo-substituted, as seen in the comparison of **1d** (R = 4-Cl) *vs*. **1a** (R = 4-Br), **3b** (R = 4-Cl) *vs*. **3e** (R = 4-Br), **5c** (R = 4-Cl) *vs*. **5 h** (R = 4-Br), and **6c** (R = 4-Cl) *vs*. **6 h** (R = 4-Br), and within the series of R = 3-CF_3_ derivatives, the introduction of the fluoro group could make the fungicidal activity obvious improvement, as seen in the comparison of **1f** (R = 3-CF_3_–4-F) *vs*. **1e** (R = 3-CF_3_), **2e** (R = 3-CF_3_–4-F) *vs*. **2d** (R = 3-CF_3_), and **6d** (R = 3-CF_3_-4-F) *vs*. **6e** (R = 3-CF_3_). However, compounds **4c** (R = 3-CF_3_) and **4f** (R = 3-CF_3_-4-F), **5d** (R = 3-CF_3_-4-F) and **5e** (R = 3-CF_3_), **7e** (R = 3-CF_3_) and **7f** (R = 3-CF_3_-4-F) are three pairs of exceptions: **7f** exhibited weaker fungicidal activity against *Rhizoctonia solani* as compared with **7e**, whereas **4f** and **5d** showed better activity than **4c** and **5e** at 10 μg mL^−1^, respectively, and the results were just the opposite when the concentration was reduced to 1 μg mL^−1^ and 0.1 μg mL^−1^. These differences in fungicidal activity might be due to variations in combination of methoxyiminoacetate pharmacophore, pyrazole ring and aromatic ring. Moreover, the positions of substituent R have little effect on the activity, as seen in the comparison of **5b** (R = 2-CH_3_) *vs*. **5f** (R = 3-CH_3_), and **6b** (R = 2-CH_3_) *vs*. **6f** (R = 3-CH_3_).

According to the different positions of chloro group, the sequence of fungicidal activity is Cl-substituted phenyl ring > Cl-substituted pyrazole ring, as seen in the comparison of **5c** *vs*. **7a-7f**. However, compound **7a** only containing a chloro on the pyrazole ring displayed nearly equal fungicidal activity to the best **5c**, and with the increasing number of chloro group, the fungicidal activity was decreased, as seen in the comparison of **7d** *vs*. **5c** and **7a**. These observations revealed that the mono-chlorination has an important influence on the fungicidal activity.

To further investigate the effect of other halogen substituents X on the pyrazole ring, Br and I were introduced to the series of methoxyiminoacetate-derivatives, as compared with the non-substituted H and Cl. When the substituent R on the phenyl ring was the same, in most cases, the fungicidal trend of these four series against *Rhizoctonia solani* was H > Cl > Br > I. For example, within the series of R = 4-CF_3_ derivatives, compound **5e** (X = H) had better fungicidal activity than **7e** (X = Cl) and **1e** (X = Br) at 10 μg mL^−1^ and 1 μg mL^−1^, respectively, whereas **2d** (X = I) showed the weakest. Similar relationships could apply to the R = 3-CF_3_-4-F derivatives **5d** (X = H), **7f** (X = Cl), **1f** (X = Br) and **2e** (X = I), R = 4-OCF_3_ derivatives **5g** (X = H), **7c** (X = Cl), **1c** (X = Br) and **2c** (X = I), as well as R = 3-CH_3_ derivatives **5f** (X = H), **7b** (X = Cl), **1b** (X = Br) and **2a** (X = I). These results might indicate that the larger molecular volume (Br and I) was unfavorable for the intracellular uptake and transport in the fungus, and the non-substituted pyrazole ring might be the best.

In summary, the SARs study revealed that the improvement of fungicidal activity required a reasonable combination of both methoxyiminoacetate pharmacophore and electron-withdrawing substituent R, and the type and size of substituent X on the pyrazole ring were critical. The chloro group had an effective impact on the fungicidal activity whether it on the terminal phenyl ring or pyrazole ring. The present work indicated that **5c** (98.94% at 0.1 μg mL^−1^, methoxyiminoacetate pharmacophore, R = 4-Cl, X = H), **7a** (83.40% at 0.1 μg mL^−1^, methoxyiminoacetate pharmacophore, R = H, X = Cl), **6c** (71.40% at 0.1 μg mL^−1^, methoxyiminoacetamide pharmacophore, R = 4-Cl, X = H) and **3b** (65.87% at 0.1 μg mL^−1^, methoxyacrylate pharmacophore, R = 4-Cl, X = H) could be used as potential lead compounds for further studies of novel fungicides.

### Three-dimensional quantitative structure-activity relationships (3D-QSARs)

In order to obtain further insight into the structural requirements of novel strobilurin fungicides, we have performed a 3D-QSAR study of the above forty-four strobilurin analogues against *Rhizoctonia solani* using CoMFA technique. In CoMFA, it is assumed that the interaction between an analogue and its molecular target is preliminarily non-covalent and shape dependent in nature. The 3D-QSAR can be derived correlating the differences in steric and electrostatic fields surrounding a set of molecules to the fungicidal activity.

**Figure 8:**
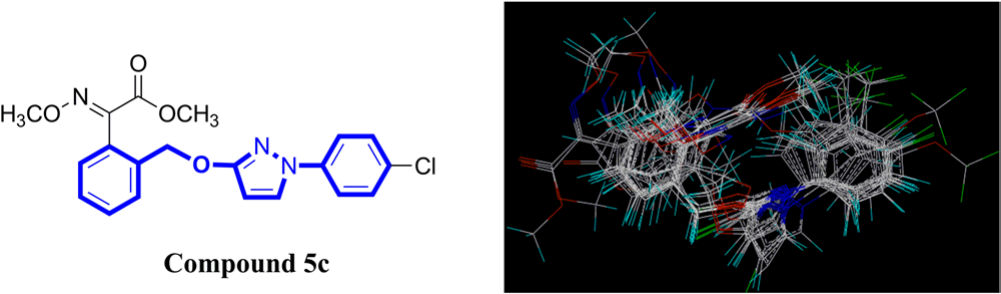
Superimposition of all molecules using database alignment.

The toxicity baselines and *EC*_50_ values were obtained from DPS data processing system, and the negative logarithm of *EC*_50_ (*pEC*_50_) was used as the biological activity in the 3D-QSAR study (Table 3). The lowest energy conformers were selected and minimized using Powell method to rms 0.001 kcal mol^−1^ Å^−1^. Alignment of the molecules was carried out using *N*-phenyl pyrazole ring as the common skeleton (Fig. 8, blue color), and the most active molecule **5c** was used as a template molecule for database alignment according to the *pEC*_50_ values. Thirty-three molecules were randomly selected as the training set to establish the CoMFA model, and the remaining eleven molecules were used as the test set to test the predictive ability of the model (Table 3, “*”). The CoMFA model was generated using Partial Least-Squares (PLS) approach. The cross-validation with the Leave-One-Out (LOO) option and the SAMPLS program was carried out to obtain the optimal number of components (*n*) and cross-validated coefficient (*q*^2^). After *n* was determined, a non-cross validated analysis was performed without column filtering to obtain regression coefficient (*R*^2^) and its standard error (SEE), as well as *F*-test value (*F*) for the model evaluation.

**Table 3:** Experimental and theoretical fungicidal activity of forty-four strobilurin analogues against *Rhizoctonia solani*.

No.	Toxicity baselines	*R* ^2^	*EC*_50_ (μM)	*pEC*_50_	Predicted *pEC*_50_	No.	Toxicity baselines	*R* ^2^	*EC*_50_ (μM)	*pEC*_50_	Predicted *pEC*_50_
**1a**	Y = 0.1548 × +5.0705	0.9653	0.670	6.174	6.832	5a*	Y = 0.1195 × +4.9791	0.9994	4.094	5.388	6.189
**1b**	Y = 0.1680 × +4.8752	0.9873	12.075	4.918	4.776	**5b**	Y = 0.0808 × +5.0415	0.9330	0.808	6.093	6.150
**1c**	Y = 0.1344 × +4.9099	0.9902	8.864	5.052	4.980	**5c**	Y = 1.3478 × +9.1015	0.8660	0.00225	8.648	8.659
**1d**	Y = 0.1469 × +5.3148	0.9916	0.015	7.823	7.272	**5d**	Y = 0.6932 × +5.6521	0.9992	0.254	6.595	6.506
1e*	Y = 0.1859 × +4.8678	0.9977	10.038	4.998	4.240	**5e**	Y = 0.5458 × +5.6282	0.9242	0.163	6.788	6.714
**1f**	Y = 0.1983 × +4.9448	0.9945	3.581	5.446	5.427	**5 f**	Y = 0.1134 × +4.9905	0.9994	3.196	5.495	5.470
**2a**	Y = 0.2308 × +4.5577	0.9971	163.403	3.787	3.768	**5 g**	Y = 0.1877 × +5.0511	0.9998	1.189	5.925	5.874
2b*	Y = 0.2595 × +5.0316	0.9951	1.224	5.912	5.931	**5 h**	Y = 0.1375 × +5.0934	0.9998	0.471	6.679	6.814
**2c**	Y = 0.1712 × +4.8075	0.9728	23.161	4.635	4.724	6a*	Y = 0.2084 × +4.8674	0.9808	11.881	4.925	5.212
**2d**	Y = 0.1355 × +4.8334	0.9957	30.306	4.519	4.556	**6b**	Y = 0.1480 × +5.0480	0.9802	1.257	5.901	5.904
**2e**	Y = 0.1067 × +5.0216	0.9176	1.086	5.964	5.971	**6c**	Y = 2.2174 × +7.3938	0.9569	0.209	6.680	6.744
**3a**	Y = 0.1329 × +4.8697	0.9574	26.246	4.581	4.623	**6d**	Y = 1.0536 × +6.1433	0.9885	0.183	6.739	6.792
3b*	Y = 0.3308 × +5.784	0.9741	0.0108	7.967	7.663	**6e**	Y = 0.3965 × +5.0009	0.9918	2.300	5.638	5.639
3c*	Y = 0.1754 × +4.9515	0.9966	4.994	5.302	5.460	**6 f**	Y = 0.0871 × +5.0552	0.7815	0.614	6.212	6.184
3d*	Y = 0.2636 × +4.7920	0.9950	14.222	4.847	3.969	6 g*	Y = 0.2434 × +4.8629	0.9972	8.157	5.089	4.879
**3e**	Y = 0.1468 × +5.0498	0.9921	1.034	5.986	5.933	**6 h**	Y = 0.2662 × +4.9499	0.9988	3.481	5.458	5.378
4a*	Y = 0.3140 × +4.6536	0.9910	33.427	4.476	4.672	**7a**	Y = 2.0149 × +7.6913	0.9696	0.116	6.937	6.808
**4b**	Y = 0.4655 × +4.4200	0.8782	44.074	4.356	4.333	**7b**	Y = 0.1523 × +4.9041	0.9463	10.305	4.987	5.139
4c*	Y = 0.2064 × +4.6537	0.9962	109.776	3.960	4.074	**7c**	Y = 0.3146 × +4.8637	0.9859	5.604	5.252	5.233
**4d**	Y = 0.1482 × +4.6063	0.9935	1009.92	2.996	2.881	**7d**	Y = 0.5507 × +5.6712	0.9402	0.139	6.857	6.808
**4e**	Y = 0.5148 × +4.5699	0.9996	17.124	4.766	4.825	7e*	Y = 0.4260 × +5.5412	0.9663	0.115	6.940	6.297
**4 f**	Y = 0.3469 × +4.6395	0.9273	24.248	4.615	4.673	**7f**	Y = 0.3960 × +4.6790	0.9299	13.307	4.874	4.950
						***pyraclostrobin***	Y = 0.3292 × +4.8818	0.9187	5.896	5.229	5.254

**Figure 9:**
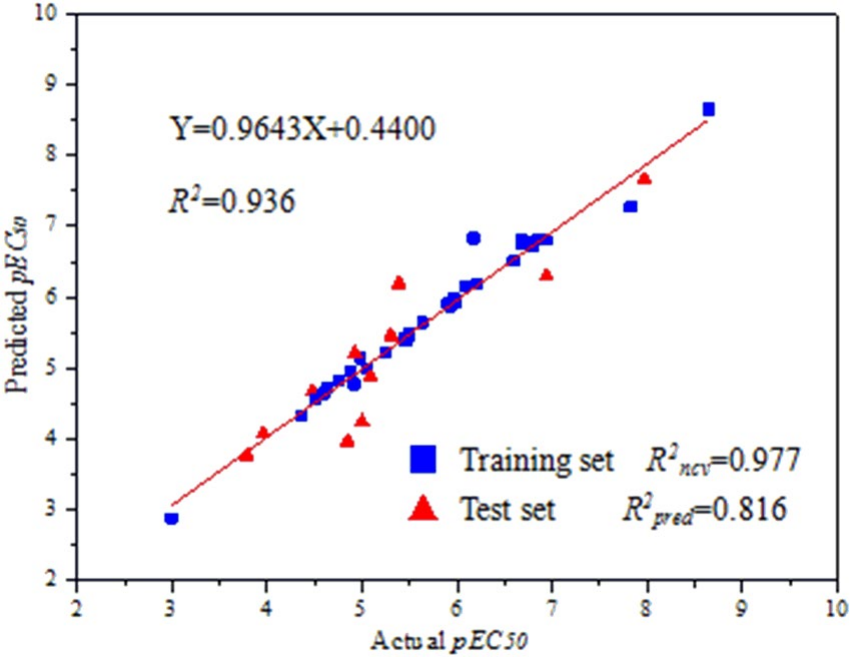
Observed (X-axis) and predicted (Y-axis) biological activities of the training (blue dot) and test (red dot) sets. (Thirty-three molecules were randomly selected as the training set and eleven molecules (Table 3, “*”) were used as the test set to test the predictive ability of the model. The observed and predicted *pEC*_50_ could be obtained according to the location of each dot; the values were listed in Table 3).

The alignment gave a conventional *R*^2^ (*R*^2^_*ncv*_) of 0.977 with 2 components, a predictive *R*^2^ (*R*^2^_*pred*_) of 0.816 and an *F* value of 94.553. The model generated with a good internal predictive ability (*q*^2^ = 0.508) and a small standard error of estimation (SEE = 0.202) was selected as the best model to explain SARs and carry out further analysis. Observed and predicted fungicidal activity of the training and test sets were plotted in Fig. 9 and listed in Table 3. The results indicated that the observed and predicted data were in good agreement with each other, with a correlation coefficient *R*^2^ of 0.936.

**Figure 10:**
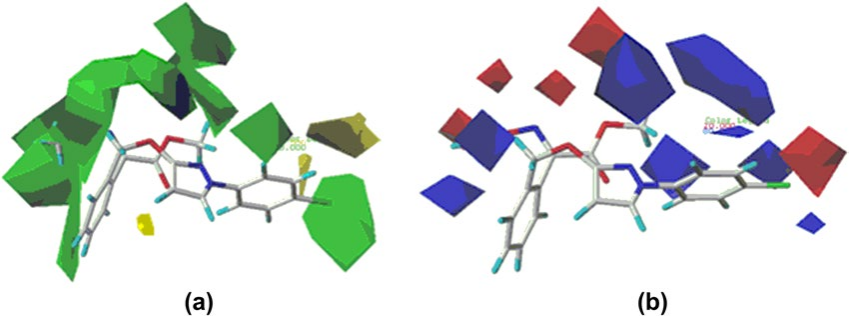
CoMFA steric (**a**) and electrostatic (**b**) contour plots.

Figure 10a displayed the steric contour plot. The green contours describe regions where sterically favorable groups enhance activity (80% contribution), and yellow contours describe regions of unfavorable steric effects (20% contribution). The pharmacophore and its linked phenyl ring were surrounded by the sterically favorable green contours. The most active molecule **5c** had a methoxyiminoacetate moiety substituted phenyl ring embedded in this green region. Other sterically-favorable green contours were observed near the terminal phenyl ring. This green contour was surrounded by the unfavorable yellow region. A substitution on the 2- or 4-position of the terminal phenyl ring was favored, whereas any substitution on the adjacent 3- or 5-position was unfavorable. This also suggested that the introduction of the chloro group to the 4-position was important for fungicidal activity.

Figure 10b displayed the electrostatic contour plot. The blue contours describe regions where positively charged groups enhance activity (80% contribution), and red contours describe regions where negatively charged groups enhance the activity (20% contribution). In compound **5c**, the red contours were found near the methoxyiminoacetate pharmacophore and the 4-position of the terminal phenyl ring, suggesting that a high electron density in this region increased the activity. A large negative-charge unfavorable blue contour was found to surround the side chain CH_2_ and the terminal phenyl ring. This indicated that substitutions in these regions with high electron density reduced activity and emphasized the necessity of positively charged groups. Overall, steric interactions (56.3% contribution) played a major role in the influence of fungicidal activity than electrostatic interactions (43.7% contribution).

### Density functional theory (DFT) calculation

The frontier-orbital energies of a compound play an important role in bioactivities^20^. E_HOMO_ is a rough measure of the electron-donating ability of a compound and, normally, increasing its value can improve the biological activity, whereas the E_LUMO_ acts in reverse^21,22^. Energy gap between HOMO and LUMO characterizes the molecular chemical stability and it is a critical parameter in determining molecular electrical transport properties because it is a measure of electron conductivity. It also affects the bioactivity of a compound. Thus, the study of the frontier-orbital energies may be helpful to the investigation of fungicidal activity. Compounds **5c**, **7a** and pyraclostrobin which had wide difference in activity were selected for DFT comparison.

**Figure 11:**
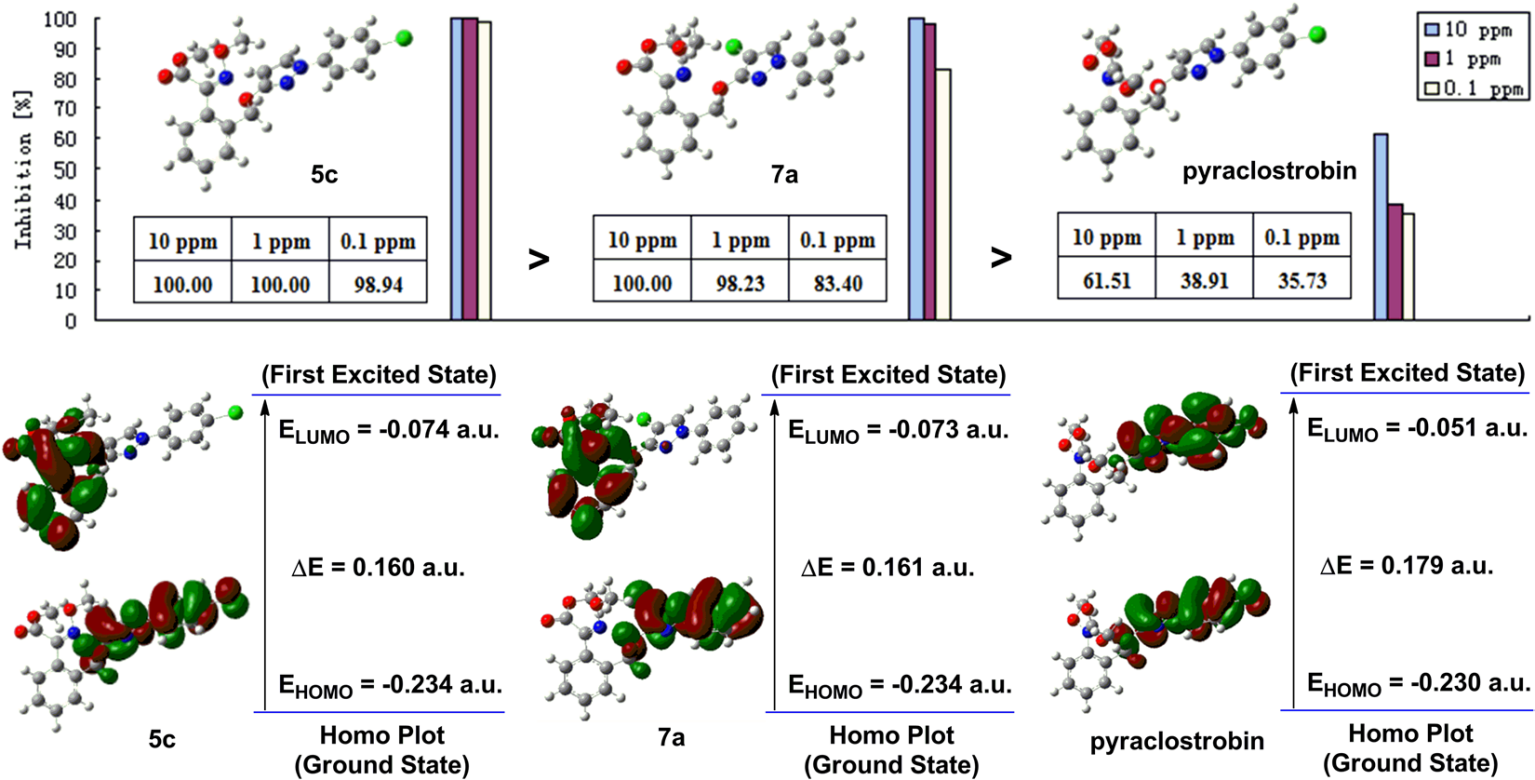
Fungicidal activity and DFT comparison of **5c**, **7a** and pyraclostrobin. (The DFT calculations were carried out in the ground-state (*in vacuo*) with Gaussian 09 software by using B3LYP/6–31 G* method. The HOMO and LUMO maps were extracted from GuassView 5.0 program based on the optimized structures).

The LUMO and HOMO maps of **5c**, **7a** and pyraclostrobin were shown in Fig. 11. Comparing the HOMO-LUMO gaps of the three molecules, the order was: pyraclostrobin >**7a** > **5c**. The narrow HOMO-LUMO gap implies a high chemical reactivity because it is energetically favorable to add electrons to a low-lying LUMO or extract electrons from a high-lying HOMO, and so to form an activated complex in any potential reaction^23^. This suggested that compound **5c** might possess a relatively high activity, which correlated well with the fungicidal activity results. In addition, the calculations indicated some similarities between **5c** and **7a**. In the HOMO, the electrons were mainly delocalized on the terminal benzene ring and pyrazole ring (including CH_2_, O and Cl atoms). When electron transitions took place, some electrons in the HOMO would enter into the LUMO^24^; then, in the LUMO, the electrons were similarly delocalized on the bridge benzene ring and oxime ester moiety. The HOMO-LUMO gaps of **5c** (0.160 a.u) and **7a** (0.161 a.u) were very close to each other. The similar electron distributions and energy gaps between **5c** and **7a** might cause both with excellent fungicidal activity. However, pyraclostrobin exhibited quite different electron distributions as compared with **5c** and **7a**. Whether in the HOMO or LUMO, its electrons were mainly delocalized on the terminal benzene ring and pyrazole ring. In the LUMO maps, the general trend of electron delocalization was: pyraclostrobin >**7a** > **5c**, which represented a negative correlation with their fungicidal activity. As reported, the frontier molecular orbitals are located on the main groups, the atoms of which can easily bind with the receptor^12,13^. Moreover, the different degrees of delocalization may affect the orbital interaction^25^. Therefore, it seemed that the high electron delocalization of pyraclostrobin in the LUMO might potentially make the orbital interactions limited, which might lead to a decrease in activity.

**Figure 12:**
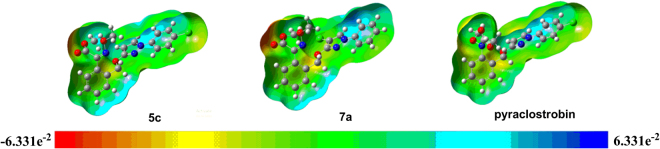
The molecular electrostatic potential (MEP) of **5c**, **7a** and pyraclostrobin. (The calculations were carried out in the ground-state (*in vacuo*) with Gaussian 09 software by using B3LYP/6–31 G* method. The MEP was extracted from GuassView 5.0 program based on the optimized structures).

Figure 12 is the molecular electrostatic potential (MEP) of **5c**, **7a** and pyraclostrobin. The MEP simultaneously displays molecular size, shape as well as positive, negative and neutral electrostatic potential regions in terms of color grading. From MEP, we can know the rich electron region and the lack electron region, where potential increases in the order of red < orange < yellow < green < blue. As can be seen from the MEP of **5c** and **7a**, the carbonyl oxygen atom on the methoxyiminoacetate pharmacophore had the greatest negative charges. Thus, it seemed probable that the oxygen atom interacted with the receptor.

## Experimental

### Chemical synthesis

All reagents were used in analytical grades. All reaction were monitored by thin layer chromatography (TLC), visualization was effected by UV (254 nm). Column chromatography was performed on flash silica gel (300–400 mesh) using mixtures of petroleum ether with ethyl acetate as eluent. Melting points were measured on an X-4 microscope electrothermal apparatus (Taike China) and were uncorrected. NMR spectra were recorded on a Bruker AV-400 spectrometer (^1^H NMR at 400 Hz, ^13^C NMR at 100 Hz) in deuterated solvents using TMS as an internal standard. Chemical shifts (*δ*) were given in parts per million (ppm), and coupling constants (*J*) were given in Hertz (Hz). The synthetic procedures and detailed characterization data of intermediates **I**, **II**, **III**, **IV** and **5** can be found in the ESI.

### General procedure for the synthesis of products 1a-1f and 3a-3e

To a solution of **IV** or **I** (1.0 mmol) in acetone (30 mL) was added K_2_CO_3_ (1.5 mmol). The mixture was refluxed for 15 min, then **II** or **III** (1.1 mmol) was added slowly. The mixture was refluxed for about 4 h (monitored by TLC), then K_2_CO_3_ was filtered off. The solvent was evaporated under reduced pressure. The crude product was purified by flash column chromatography on silica gel (eluent for **1a-1f**: ethyl acetate/petroleum ether, 1: 5v/v; for **3a-3e**: ethyl acetate/petroleum ether, 1: 8v/v) to afford products.

#### (*E*)-Methyl 2-(2-(((4-bromo-1-(4-bromophenyl)-1*H*-pyrazol-3-yl)oxy)methyl)phenyl)-2-(methoxyimino)acetate (1a)

White solid; Yield 80%; m.p. 154–155 °C; ^1^H NMR (400 MHz, CDCl_3_) *δ* 7.66 (s, 1H, pyrazole-H), 7.65–7.13 (m, 8H, Ar-H), 5.16 (s, 2H, CH_2_), 3.97 (s, 3H, OCH_3_), 3.77 (s, 3H, OCH_3_); ^13^C NMR (100 MHz, CDCl_3_) *δ* 162.2, 159.4, 148.3, 137.6, 133.7, 131.4, 128.9, 128.4, 127.7, 127.4, 127.0, 126.7, 117.9, 81.9, 68.5, 62.8, 52.0.

#### (*E*)-Methyl 2-(2-(((4-bromo-1-(*m*-tolyl)-1*H*-pyrazol-3-yl)oxy)methyl)phenyl)-2-(methoxyimino)acetate (1b)

White solid; Yield 81%; m.p. 134–135 °C; ^1^H NMR (400 MHz, CDCl_3_) *δ* 7.73 (s, 1H, pyrazole-H), 7.64–7.01 (m, 8H, Ar-H), 5.25 (s, 2H, CH_2_), 4.04 (s, 3H, OCH_3_), 3.83 (s, 3H, OCH_3_), 2.39 (s, 3H, CH_3_); ^13^C NMR (100 MHz, CDCl_3_) *δ* 163.3, 160.2, 149.4, 139.7, 134.9, 130.0, 129.4, 129.2, 128.8, 128.4, 128.0, 127.9, 126.6, 118.4, 114.8, 82.0, 69.5, 63.8, 60.4, 53.1, 21.5.

#### (*E*)-Methyl 2-(2-(((4-bromo-1-(4-(trifluoromethoxy)phenyl)-1*H*-pyrazol-3-yl)oxy)methyl)phenyl)-2-(methoxyimino)acetate (1c)

White solid; Yield 78%; m.p. 154–155 °C; ^1^H NMR (400 MHz, CDCl_3_) *δ* 7.56 (s, 1H, pyrazole-H), 7.54–7.13 (m, 8H, Ar-H), 5.16 (s, 2H, CH_2_), 3.98 (s, 3H, OCH_3_), 3.78 (s, 3H, OCH_3_); ^13^C NMR (100 MHz, CDCl_3_) *δ* 163.3, 160.5, 149.3, 138.2, 134.8, 130.0, 129.5, 128.8,128.5, 128.1, 127.9, 122.2, 118.7, 83.1, 69.5, 63.9, 53.1.

#### (*E*)-Methyl 2-(2-(((4-bromo-1-(4-chlorophenyl)-1*H*-pyrazol-3-yl)oxy)methyl)phenyl)-2-(methoxyimino)acetate (1d)

White solid; Yield 85%; m.p. 147–148 °C; ^1^H NMR (400 MHz, CDCl_3_) *δ* 7.65 (s, 1H, pyrazole-H), 7.55–7.13 (m, 8H, Ar-H), 5.16 (s, 2H, CH_2_), 3.97 (s, 3H, OCH_3_), 3.78 (s, 3H, OCH_3_); ^13^C NMR (100 MHz, CDCl_3_) *δ* 163.3, 160.4, 149.3, 138.2, 134.8, 131.1, 130.0, 129.5, 128.8, 128.5, 128.1, 127.8, 118.7, 82.9, 69.5, 63.9, 53.1.

#### (*E*)-Methyl 2-(2-(((4-bromo-1-(3-(trifluoromethyl)phenyl)-1*H*-pyrazol-3-yl)oxy)methyl)phenyl)-2-(methoxyimino)acetate (1e)

White solid; Yield 83%; m.p. 122–123 °C; ^1^H NMR (400 MHz, CDCl_3_) *δ* 7.74 (s, 1H, pyrazole-H), 7.63–7.13 (m, 8H, Ar-H), 5.18 (s, 2H, CH_2_), 3.98 (s, 3H, OCH_3_), 3.78 (s, 3H, OCH_3_). ^13^C NMR (100 MHz, CDCl_3_) *δ* 163.3, 160.7, 149.3, 139.9, 134.6, 130.1, 130.0, 129.5, 128.9, 128.5, 128.2, 127.9, 122.1, 120.2, 114.4, 83.6, 69.7, 63.9, 53.1.

#### (*E*)-Methyl 2-(2-(((4-bromo-1-(4-fluoro-3-(trifluoromethyl)phenyl)-1*H*-pyrazol-3-yl)oxy)methyl)phenyl)-2-(methoxyimino)acetate (**1f**)

White solid; Yield 84%; m.p. 168–169 °C; ^1^H NMR (400 MHz, CDCl_3_) *δ* 7.70 (q, 1H, Ar-H), 7.67 (s, 1H, pyrazole-H), 7.61–7.13 (m, 7H, Ar-H), 5.16 (s, 2H, CH_2_), 3.97 (s, 3H, OCH_3_), 3.78 (s, 3H, OCH_3_); ^13^C NMR (100 MHz, CDCl_3_) *δ* 163.3, 160.7, 149.3, 135.8, 134.6, 129.5, 128.9, 128.5, 128.2, 122.4, 122.3, 118.2, 117.9, 83.62, 69.7, 63.9, 53.1.

#### (*E*)-Methyl 3-methoxy-2-(2-(((1-phenyl-1*H*-pyrazol-3-yl)oxy)methyl)phenyl)acrylate (3a)

White solid; Yield 75%; m.p. 100–101 °C; ^1^H NMR (400 MHz, CDCl_3_) *δ* 7.62 (d, *J* = 2.8 Hz, 1H, pyrazole-H), 7.59 (d, *J* = 8.4 Hz, 1H, Ar-H), 7.54 (s, 1H, CH-OCH_3_), 7.52–7.08 (m, 8H, Ar-H), 5.77 (d, *J* = 2.8 Hz, 1H, pyrazole-H), 5.12 (s, 2H, CH_2_), 3.71 (s, 3H, OCH_3_), 3.60 (s, 3H, OCH_3_); ^13^C NMR (100 MHz, CDCl_3_) *δ* 168.1, 164.5, 160.2, 140.2, 136.2, 131.8, 131.0, 129.3, 128.2, 128.0, 127.7, 125.2, 117.8, 110.1, 94.0, 68.9, 62.0, 51.7.

#### (*E*)-Methyl 2-(2-(((1-(4-chlorophenyl)-1*H*-pyrazol-3-yl)oxy)methyl)phenyl)-3-methoxyacrylate (3b)

White solid; Yield 78%; m.p. 95–96 °C; ^1^H NMR (400 MHz, CDCl_3_) *δ* 7.60 (d, *J* = 2.4 Hz, 1H, pyrazole-H), 7.55 (d, *J* = 8.8 Hz, 1H, Ar-H), 7.53 (s, 1H, CH- OCH_3_), 7.48–7.10 (m,7H, Ar-H), 5.80 (d, *J* = 2.4 Hz, 1H, pyrazole-H), 5.11 (s, 2H, CH_2_), 3.74 (s, 3H, OCH_3_), 3.62 (s, 3H, OCH_3_); ^13^C NMR (100 MHz, CDCl_3_) *δ* 168.0, 164.6, 160.2, 138.8, 136.0, 131.7, 131.0, 130.5, 129.4, 128.2, 128.0, 127.7, 127.6, 118.9, 110.1, 94.6, 68.9, 62.0, 51.7.

#### (*E*)-Methyl 3-methoxy-2-(2-(((1-(*o*-tolyl)-1*H*-pyrazol-3-yl)oxy)methyl)phenyl)acrylate (3c)

White solid; Yield 72%; m.p. 115–116 °C; ^1^H NMR (400 MHz, CDCl_3_) *δ* 7.54 (d, *J* = 2.4 Hz, 1H, pyrazole-H), 7.48 (s, 1H, CH-OMe), 7.28–7.07 (m, 8H, Ar-H), 5.73 (d, *J* = 2.4 Hz, 1H, pyrazole-H), 5.08 (s, 2H, CH_2_), 3.67 (s, 3H, OCH_3_), 3.57 (s, 3H, OCH_3_), 2.20 (s, 3H, CH_3_); ^13^C NMR (100 MHz, CDCl_3_) *δ* 167.0, 163.0, 159.1, 130.7, 130.4, 129.9, 127.2, 126.9, 126.7, 126.6, 125.5, 124.7, 109.1, 91.2, 67.8, 60.9, 50.6, 17.3.

#### (*E*)-Methyl 3-methoxy-2-(2-(((1-(3-(trifluoromethyl)phenyl)-1*H*-pyrazol-3-yl)oxy)methyl)phenyl)acrylate (3d)

White solid; Yield 73%; m.p. 108–109 °C; ^1^H NMR (400 MHz, CDCl_3_) *δ* 7.80 (d, *J* = 2.4 Hz, 1H, pyrazole-H), 7.67 (d, 2H, Ar-H), 7.52 (s, 1H, CH-OCH_3_), 7.43–7.12 (m,6H, Ar-H), 5.83 (d, *J* = 2.4 Hz, 1H, pyrazole-H), 5.14 (s, 2H, CH_2_), 3.73 (s, 3H, OCH_3_), 3.61 (s, 3H, OCH_3_); ^13^C NMR (100 MHz, CDCl_3_) *δ* 167.8, 163.7, 159.3, 138.3, 135.8, 131.5, 131.1, 130.9, 130.5, 129.5, 128.3, 128.1, 127.8, 127.3, 118.2, 110.1, 94.5, 69.0, 62.0, 51.6.

#### (*E*)-Methyl 2-(2-(((1-(4-bromophenyl)-1*H*-pyrazol-3-yl)oxy)methyl)phenyl)-3-methoxyacrylate (3e)

White solid; Yield 75%; m.p. 96–97 °C; ^1^H NMR (400 MHz, CDCl_3_) *δ* 7.60 (d, *J* = 2.4 Hz, 1H, pyrazole-H), 7.55 (d, 1H, Ar-H), 7.52 (s, 1H, CH-OCH_3_), 7.45–7.09 (m,7H, Ar-H), 5.80 (d, *J* = 2.4 Hz, 1H, pyrazole-H), 5.11 (s, 2H, CH_2_), 3.73 (s, 3H, OCH_3_), 3.62 (s, 3H, OCH_3_); ^13^C NMR (100 MHz, CDCl_3_) *δ* 168.0, 164.6, 160.2, 139.2, 136.0, 132.3, 131.7, 131.0, 128.2, 128.0, 127.7, 127.6, 119.1, 118.1, 110.1, 94.6, 69.0, 62.0, 51.7.

### General procedure for the synthesis of products 2a-2e

Compound **5** (5 mmol) was dissolved in CHCl_3_ (30 mL), then K_2_CO_3_ (10 mmol) and ICl (10 mmol) were added slowly. The reaction mixture was stirred at 20 °C for about 5 h (monitored by TLC). The precipitate was filtered off, and the solvent was evaporated under reduced pressure. The residue was quenched with Na_2_S_2_O_3_, and extracted with ethyl acetate, dried, filtered, and evaporated. The crude product was purified by flash column chromatography on silica gel (eluent: ethyl acetate/petroleum ether, 1: 4 v/v) to afford products **2a-2e**.

#### (*E*)-Methyl 2-(2-(((4-iodo-1-(*m*-tolyl)-1*H*-pyrazol-3-yl)oxy)methyl)phenyl)-2-(methoxyimino)acetate (2a)

White solid; Yield 82%; m.p. 166–167 °C; ^1^H NMR (400 MHz, CDCl_3_) *δ* 7.81 (s, 1H, pyrazole-H), 7.79–7.06 (m, 8H, Ar-H), 5.26 (s, 2H, CH_2_), 4.08 (s, 3H, OCH_3_), 3.89 (s, 3H, OCH_3_), 2.49 (s, 3H, CH_3_); ^13^C NMR (100 MHz, CDCl_3_) *δ* 163.3, 163.2, 149.3, 142.7, 139.7, 139.6, 135.0, 132.0, 129.8, 129.5, 128.6, 128.5, 128.0, 96.5, 69.5, 63.9, 53.2, 47.2, 28.3.

#### (*E*)-Methyl 2-(2-(((4-iodo-1-(4-iodophenyl)-1*H*-pyrazol-3-yl)oxy)methyl)phenyl)-2-(methoxyimino)acetate (2b)

White solid; Yield 80%; m.p. 82–83 °C; ^1^H NMR (400 MHz, CDCl_3_) *δ* 7.76 (s, 1H, pyrazole-H), 7.74–7.23 (m, 8H, Ar-H), 5.25 (s, 2H, CH_2_), 4.08 (s, 3H, OCH_3_), 3.89 (s, 3H, OCH_3_); ^13^C NMR (100 MHz, CDCl_3_) *δ* 163.4, 163.2, 149.3, 139.3, 138.3, 134.9, 131.9, 129.8, 129.5, 128.6, 128.5, 128.0, 119.3, 89.4, 69.5, 63.9, 53.2, 47.5.

#### (*E*)-Methyl 2-(2-(((4-iodo-1-(4-(trifluoromethoxy)phenyl)-1*H*-pyrazol-3-yl)oxy)methyl)phenyl)-2-(methoxyimino)acetate (2c)

White solid; Yield 83%; m.p. 164–165 °C; ^1^H NMR (400 MHz, CDCl_3_) *δ* 7.65 (s, 1H, pyrazole-H), 7.56–7.12 (m, 8H, Ar-H), 5.15 (s, 2H, CH_2_), 3.97 (s, 3H, OCH_3_), 3.77 (s, 3H, OCH_3_); ^13^C NMR (100 MHz, CDCl_3_) *δ* 163.5, 163.3, 149.3, 146.7, 146.6, 138.2, 134.9, 130.0, 129.5, 128.6, 128.5, 128.0, 122.2, 118.8, 69.5, 63.9, 53.2, 47.5.

#### (E)-Methyl 2-(2-(((4-iodo-1-(3-(trifluoromethyl)phenyl)-1H-pyrazol-3-yl)oxy)methyl)phenyl)-2-(methoxyimino)acetate (2d)

White solid; Yield 88%; m.p. 124–125 °C; ^1^H NMR (400 MHz, CDCl_3_) *δ* 7.74 (s, 1H, pyrazole-H), 7.63–7.13 (m, 8H, Ar-H), 5.18 (s, 2H, CH_2_), 3.98 (s, 3H, OCH_3_), 3.79 (s, 3H, OCH_3_); ^13^C NMR (100 MHz, CDCl_3_) *δ* 163.6, 163.3, 149.3, 140.0, 134.8, 132.1, 130.1, 129.8, 129.5, 128.7, 128.5, 128.0, 122.1, 120.3, 114.4, 69.7, 63.9, 53.2, 48.1.

#### (E)-Methyl 2-(2-(((1-(4-fluoro-3-(trifluoromethyl)phenyl)-4-iodo-1H-pyrazol-3-yl)oxy)methyl)phenyl)-2-(methoxyimino)acetate (2e)

White solid; Yield 86%; m.p. 101–102 °C; ^1^H-NMR (400 MHz, CDCl_3_) *δ* 7.80 (m, 1H, Ar-H), 7.77 (s, 1H, pyrazole-H), 7.73–7.23 (m, 6H, Ar-H), 5.26 (s, 2H, CH_2_), 4.09 (s, 3H, OCH_3_), 3.90 (s, 3H, OCH_3_); ^13^C NMR (100 MHz, CDCl_3_) *δ* 163.7, 163.3, 149.3, 135.9, 135.8, 134.7, 132.1, 129.8, 129.5, 128.7, 128.5, 128.1, 122.5, 122.4, 118.2, 117.9, 69.7, 63.9, 53.2, 48.1.

### X-ray diffraction crystallography

Suitable crystals of **1f**, **2b** and **3b** were obtained by slow evaporation of methanol solutions at r.t. Crystal data were collected on a *Nonius CAD-4* diffractometer with Mo*Kα* radiation (*λ* = 0.71073 Å) by using a *ω*/2*θ* scan mode at 293 K. The structures were solved by direct method using SHELXS-97 and refined by full-matrix least-squares procedure on *F*^2^ for all data using SHELXL-97^26^. All non-H-atoms were refined anisotropically, and the H-atoms were added at calculated positions. The isotropic temp. factors were fixed to 1.2 times (1.5 times for methyl group) the equivalent isotropic displacement parameters of the C-atom the H-atom is attached to.

CCDC-1433534, CCDC-1433532, CCDC-1433533 contain the supplementary crystallographic data for **1f**, **2b** and **3b**, respectively. These data can be obtained free of charge from the Cambridge Crystallographic Data Centre *via* http://www.ccdc.cam.ac.uk/data_request/cif (or from the CCDC, 12 Union Road, Cambridge CB2 1EZ, UK; fax: + 44 1223 3360–33; e-mail: deposit@ccdc.cam.ac.uk).

### Fungicidal activity assays

The fungicidal activity of compounds **1a-1f**, **2a-2e**, **3a-3e**, **4a-4f**, **5a-5h**, **6a-6h** and **7a-7f** against *Rhizoctonia cerealis* and *Gibberella zeae* were tested according to the literature procedures^27^. Pyraclostrobin, the lead compound, was used as a control. The results were listed in Table 3.

### 3D-QSARs details

The 3D-QSARs studies were performed using SYBYL X 2.0 software with a standard Tripos force field^28^. The compounds were constructed from the fragments in the SYBYL database with standard bond lengths and bond angles. Geometry optimization was carried out using the standard Tripos force field with distance dependent dielectric function and energy gradient of 0.001 kcal mol^−1^ Å^−1^. The lowest energy conformers were selected and minimized using the Powell method till root-mean-square (rms) deviation 0.001 kcal mol^−1^ Å^−1^ was achieved.

### DFT calculation

The DFT calculation was carried out in the ground state (*in vacuo*) with Gaussian 09 software by using B3LYP/6–31 G* method^29,30^. The geometrical, electronic and energy parameters were extracted from the Gaussian files based on the optimized structures.

## Conclusions

In summary, sixteen novel strobilurin analogues (**1a-1f**, **2a-2e**, **3a-3e**) were designed and synthesized. The structures of **1f**, **2b** and **3b** were determined by single crystal X-ray diffraction analysis. Other twenty-eight similar compounds **4a-4f**, **5a-5h**, **6a-6h** and **7a-7f** from our previous studies were also collected together with the above sixteen analogues for *in vitro* bioassays and structure-activity relationships (SARs) study in details. Most compounds exhibited excellent-to-good fungicidal activity against *Rhizoctonia solani*, especially **5c**, **7a**, **6c**, and **3b** with 98.94%, 83.40%, 71.40% and 65.87% inhibition rates at 0.1 μg mL^−1^, respectively, better than commercial pyraclostrobin. The SARs revealed that the improvement of fungicidal activity required a reasonable combination of both methoxyiminoacetate pharmacophore and electron-withdrawing substituent R, and the type and size of substituent X on the pyrazole ring was critical. The 3D-QSAR model for the above forty-four strobilurin analogues was also derived using CoMFA method, with high correlative and predictive abilities. The contour maps indicated that the electron rich substituent R on the 4-position of the terminal phenyl ring might improve activity, which was in good agreement with the SARs discussion. In addition, through DFT calculation, it seemed that the high electron delocalization of pyraclostrobin in LUMO might possibly make the orbital interactions limited, which might bring out a decrease in activity, whereas the similar electron distributions and narrow energy gaps between **5c** and **7a** might give both with excellent fungicidal activity. The present work indicated that **5c**, **7a**, **6c** and **3b** could be used as potential lead compounds for further studies of novel fungicides.

## Electronic supplementary material

Supporting Information
