# Supplementary material for: Synthesis, fungicidal activity, structure-activity relationships (SARs) and density functional theory (DFT) studies of novel strobilurin analogues containing arylpyrazole rings
Source: Sci Rep. 2018 May 18;8:7822. doi: 10.1038/s41598-018-26154-5 (PMC5959921; doi:10.1038/s41598-018-26154-5)
Supplement: Supplementary file 1 — Supporting Information [file 41598_2018_26154_MOESM1_ESM.pdf]

Electronic Supplementary Information (ESI) for

Synthesis, fungicidal activity, structure-activity relationships (SARs) and density functional theory (DFT) studies of novel strobilurin analogues containing arylpyrazole rings

Yuanyuan Liu,*[a] Kunzhi Lv,[b] Yi Li,*[c] Qiuli Nan[a] and Jinyuan Xu[c]

[a] *Department of Chemical and Pharmaceutical Engineering, Southeast University ChengXian College, Nanjing 210088, P. R. China. E-mail: liuyuanyuan1985419@163.com; Tel: +86 25 58662849*

[b] *Nanjing Sanhome Pharmaceutical Co Ltd., Nanjing 210018, P. R. China*

[c] *College of Food Science and Light Industry, Nanjing Tech University, Nanjing 211816, P. R. China. E-mail: liynj2012@njtech.edu.cn; Tel: +86 25 58139432*

Table of Contents

General procedure for the synthesis of intermediate **I**........ 2

General procedure for the synthesis of intermediate **II**........ 3

General procedure for the synthesis of intermediate III........ 4

General procedure for the synthesis of intermediate **IV**........ 6

General procedure for the synthesis of intermediate **5**........ 7

X-Ray Crystallographic Analysis of **1f**, **2b** and **3b**........ 10

General procedure for the synthesis of intermediate I

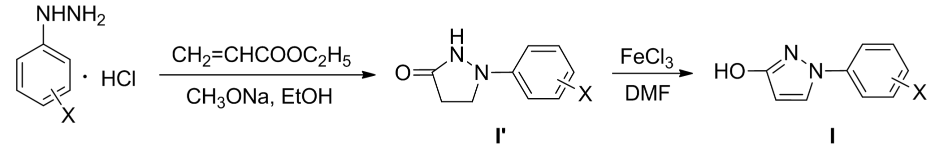

a: R = 4-Br **b**: R = 3-CH_3 **c**: R = 4-OCF_3 **d**: R = 4-Cl **e**: R = 3-CF_3 **f**: R = 3-CF_3-4-F

Intermediate *N*-arylpyrazole **I** was synthesized from arylhydrazines *via* addition-cyclization and oxidation, according to the reported methods[1].

Compound **I'**: Ethyl acrylate (0.45 mol) was added dropwise at 40-45°C within 1 h to a mixture of sodium methoxide (0.2 mol), ethanol (50 mL), toluene (55 mL) and aryl hydrazine hydrochloride (0.09 mol). The mixture was subsequently stirred for 15 h at 45°C, and then evaporated to 40 mL. The residue was taken up in sufficient water, and washed with toluene. The organic phases were extracted with 5% NaOH solution. The aqueous phases were adjusted to pH 6.5 and cooled to 5°C. The solid formed was filtered off, washed with water and dried to afford **I'**.

Compound **I**: Using oxygen as oxidizing agent, compounds **I'** (150 mmol) was dissolved in DMF (100 mL) and mixed with $FeCl_3{\cdot}6H_2O$ (2.4 g, 15 mmol). The mixture was heated to 80°C and maintained at that temperature for 4 h, and then stirred at 30°C for another 20 h. The reaction mixture was then poured into water (500 mL) with good stirring. The precipitate which formed was filtered off, washed with H_2O and dried to afford **I**.

1-(4-Bromophenyl)-1*H*-pyrazol-3-ol (Ia)

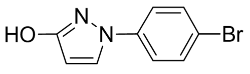

Red solid (yield: 70%); m.p. 220-222°C; ^{1}H NMR (400 MHz, DMSO-d_6) δ 10.18 (s, 1H, OH), 8.15 (d, J = 2.2 Hz, 1H, pyrazole-H), 7.71-7.28 (m, 4H, Ar-H), 5.79 (d, J = 2.2 Hz, 1H, pyrazole-H).

1-(*m*-Tolyl)-1*H*-pyrazol-3-ol (Ib)

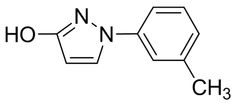

Red solid (yield: 75%); m.p. 128-130°C; ^{1}H NMR (400 MHz, DMSO-d_6) δ 10.02 (s, 1H, OH), 7.71 (d, J = 2.2 Hz, 1H, pyrazole-H), 7.31 (m, 4H, Ar-H), 5.74 (d, J = 2.2 Hz, 1H, pyrazole-H), 2.27 (s, 3H, CH_3).

1-(4-(Trifluoromethoxy)phenyl)-1*H*-pyrazol-3-ol (Ic)

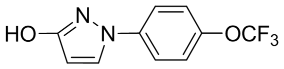

Gray solid (yield: 80%); m.p. 189-191°C; ^{1}H NMR (400 MHz, DMSO-d_6) δ 10.41 (s, 1H, OH), 8.26 (d, J = 2.4 Hz, 1H, pyrazole-H), 7.79 (d, J = 7.6 Hz, 2H, Ar-H), 7.44 (d, J = 7.2 Hz, 2H, Ar-H), 5.86 (d, J = 2.4 Hz, 1H, pyrazole-H).

1-(4-Chlorophenyl)-1*H*-pyrazol-3-ol (Id)

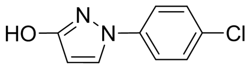

Red solid (yield: 78%); m.p. 189-191°C； ^{1}H NMR (400 MHz, DMSO-d_6) δ 10.37 (s, 1H, OH), 8.24 (d, J = 2.4 Hz, 1H, pyrazole-H), 7.70 (d, J = 8.4 Hz, 2H, Ar-H), 7.48 (d, J = 8.4 Hz, 2H, Ar-H), 5.84 (d, J = 2.4 Hz, 1H, pyrazole-H).

1-(3-(Trifluoromethyl)phenyl)-1*H*-pyrazol-3-ol (Ie)

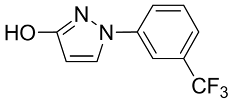

Gray solid (yield: 72%); m.p. 121-123°C; ^{1}H NMR (400 MHz, DMSO-d_6) δ 10.38 (s, 1H, OH), 8.34 (d, J = 2.5 Hz, 1H, pyrazole-H), 7.95 (s, 1H, Ar-H), 7.61-7.48 (s, 3H, Ar-H), 5.83 (d, J = 2.5 Hz, 1H, pyrazole-H).

1-(4-Fluoro-3-(trifluoromethyl)phenyl)-1*H*-pyrazol-3-ol (If)

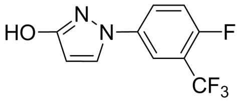

Gray solid (yield: 72%); m.p. 129-131°C; ^{1}H NMR (400 MHz, DMSO-d_6) δ 10.50 (s, 1H, OH), 8.36 (d, J = 2.5 Hz, 1H, pyrazole-H), 8.00 (m, 2H, Ar-H), 7.59 (s, 1H, Ar-H), 5.88 (d, J = 2.5 Hz, 1H, pyrazole-H).

General procedure for the synthesis of intermediate II

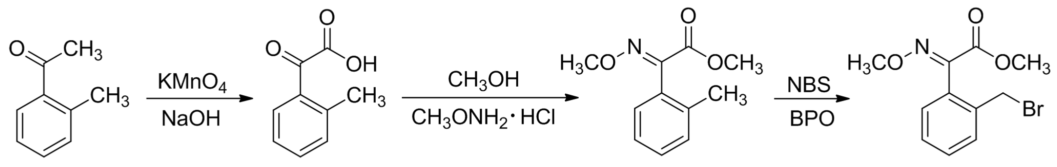

II-a **II-b** **II**

Intermediate benzyl bromide **II** was prepared from 1-(*o*-tolyl)ethanone *via* four steps including

oxidation, esterification, oximation and bromination, according to the reported methods[2].

Compound **II-a**: A mixture of 1-(*o*-tolyl)ethanone (10 g, 74.6 mmol), H_2O (150 mL), NaOH (2 g, 50 mmol) and $Bu_4N^+Br^-$ (, 0.1 g, 0.295 mmol) was stirred at 0°C, then $KMnO_4$ (23 g, 0.146 mol) was added gradually. The mixture was stirred at 30°C until the color of the solution remained unchanged. The black precipitate was filtered off, and the filtrate was neutralized by $NaHSO_3$ solution, acidified with HCl (36.5%) and filtered. The filtrate was extracted with EtOAc, and then the solvent was removed under reduced pressure to afford **II-a**.

Compound **II-b**: A mixture of **II-a** (5 g, 0.03 mol), H_2SO_4 (1 mL), and MeOH (100 mL) was heated to reflux until the starting material had been completely consumed as judged by TLC. The mixture was neutralized by $NaHCO_3$, and $CH_3ONH_2{\cdot}HCl$ (3 g, 0.036 mol) was added. The reaction was monitored by TLC, then excess MeOH was removed under reduced pressure. The residue was neutralized by $NaHCO_3$, extracted by EtOAc, dried, filtered, and evaporated under reduced pressure. It was then purified by a silica-gel column chromatography (petroleum ether/EtOAc = 8:1) to afford **II-b**.

Compound **II**: To the solution of **II-b** (5 g, 24.0 mmol) in CCl_4 (150 mL) was added NBS (4.7 g, 26.4 mmol) and BPO (1 g, 4.13 mmol). The reaction mixture was heated to reflux until the starting material had been completely consumed as judged by TLC. The precipitate was filtered off, and the solvent was evaporated under reduced pressure. The residue was purified by a silica-gel column chromatography (petroleum ether/EtOAc = 15:1) to give **II**.

(*E*)-Methyl 2-(2-(bromomethyl)phenyl)-2- (methoxyimino)acetate (II)

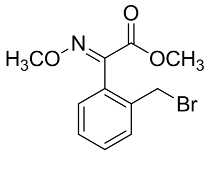

Yield 85%; Yellow oil; ^{1}H NMR (400 MHz, $CDCl_3$) δ 7.47-7.13 (m, 4H, Ar-H), 4.32 (s, 2H, CH_2), 4.04 (s, 3H, OCH_3), 3.85 (s, 3H, OCH_3).

General procedure for the synthesis of intermediate III

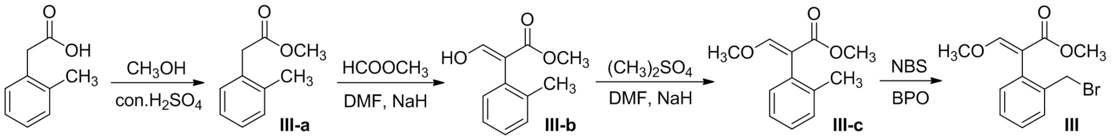

Compound **III-a**: A mixture of 2-(*o*-tolyl)acetic acid (30 mmol), H_2SO_4 (1 mL) and MeOH (50

mL) was heated to reflux until the starting material had been completely consumed as judged by TLC. The solvent was removed under reduced pressure, and water (100 mL) was added. The mixture was extracted with ethyl acetate, dried by anhydrous Na_2SO_4, and then evaporated to afford **III-a**.

Methyl 2-(*o*-tolyl)acetate (III-a)

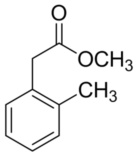

Yellow oil; Yield 90%; ^{1}H NMR (400 MHz, $CDCl_3$) δ 7.12-7.08 (m, 4H, Ar-H), 3.60 (s, 3H, OCH_3), 3.56 (s, 2H, CH_2), 2.23 (s, 3H, CH_3).

Compound **III-b**: A mixture of **III-a** (5 mmol), DMF (30 mL) and NaH (25 mmol) were stirred at 0°C for 30 min, then methyl formate (15 mmol) was added slowly. The reaction was stirred at low temperature for 30 min, and then slowly warmed up to 20°C over 12 h. Water (150 mL) was added. The mixture was acidated by HCl, extracted with CH_2Cl_2, dried by $NaSO_4$, and then evaporated to afford **III-b**.

(*E*)-Methyl 3-hydroxy-2-(*o*-tolyl)acrylate (III-b)

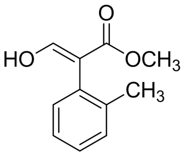

White oil; Yield 85%; ^{1}H NMR (400 MHz, $CDCl_3$) δ 11.92 (d, 1H, OH), 7.05-7.46 (m, 5H, Ar-H), 3.73 (s, 3H, OCH_3), 2.34 (s, 3H, CH_3).

Compound **III-c**: A mixture of **III-b** (10 mmol), DMF (30 mL) and NaH (20 mmol) were stirred at 20°C for 30 min, and then dimethyl sulfate (12 mmol) was added slowly. The reaction was stirred at 20°C for another 12 h, and water (100 mL) was added. The mixture was extracted with CH_2Cl_2, dried by anhydrous Na_2SO_4, and then evaporated. The residue was purified by a silica-gel column chromatography (ethyl acetate/petroleum ether, 1: 8 v/v) to give **III-c**.

(*E*)-Methyl 3-methoxy-2-(*o*-tolyl)acrylate (III-c)

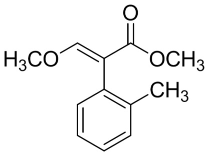

White oil; Yield 78%; ^{1}H NMR (400 MHz, $CDCl_3$) δ 7.49 (s, 1H, C=CH), 7.17-7.02 (m, 4H, Ar-H), 3.73 (s, 3H, OCH_3), 3.61 (s, 3H, $COOCH_3$), 2.11 (s, 3H, CH_3).

Compound **III**: To the solution of **III-c** (10 mmol) in CCl_4 (50 mL) was added NBS (12.5 mmol) and BPO (1 mmol). The reaction mixture was heated to reflux until the starting material had been completely consumed as judged by TLC. The precipitate was filtered off, and the solvent was evaporated under reduced pressure. The residue was purified by a silica-gel column chromatography (ethyl acetate/petroleum ether, 1: 12 v/v) to give to afford **III**.

(*E*)-Methyl 2-(2-(bromomethyl)phenyl)-3-methoxyacrylate (III)

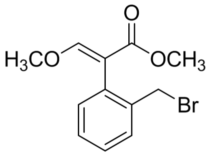

White solid; Yield 77%; m.p. 63-64°C; ^{1}H NMR (400 MHz, $CDCl_3$) δ 7.57 (s, 1H, C=CH), 7.41-7.05 (m, 4H, Ar-H), 4.34 (s, 2H, CH_2), 3.76 (s, 3H, OCH_3), 3.63 (s, 3H, $COOCH_3$).

General procedure for the synthesis of intermediate IV

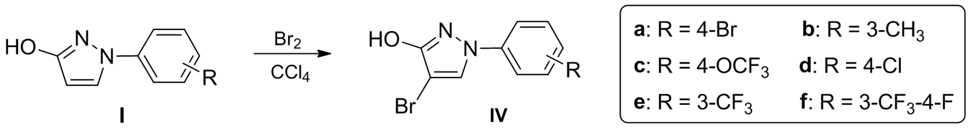

Compound **I** (5 mmol) was dissolved in CCl_4 (50 mL), then a solution of Br_2 (5 mmol) in CCl_4 (10 mL) was added slowly. The mixture was stirred at r.t. for 24 h, and then evaporated under reduced pressure. The residue was purified by a silica-gel column chromatography (ethyl acetate/petroleum ether, 1: 6 v/v) to give **IV**.

4-Bromo-1-(4-bromophenyl)-1*H*-pyrazol-3-ol (IVa)

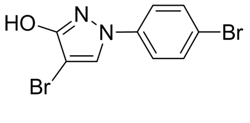

Yellow solid; Yield 75%; m.p. 200-201°C; ^{1}H NMR (400 MHz, DMSO-d_6) δ 11.07 (s,1H, OH), 8.54 (s, 1H, pyrazole-H), 7.62 (m, 4H, Ar-H).

4-Bromo-1-(*m*-tolyl)-1*H*-pyrazol-3-ol (IVb)

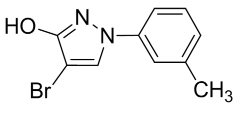

Yellow solid; Yield 78%; m.p. 203-204°C; ^{1}H NMR (400 MHz, DMSO-*d*$_6$) *δ* 7.95 (s, 1H, OH), 7.30 (s, 1H, pyrazole-H), 7.27-7.18 (m, 4H, Ar-H), 2.21 (s, 3H, Ar-CH_3).

4-Bromo-1-(4-(trifluoromethoxy)phenyl)-1*H*-pyrazol-3-ol (IVc)

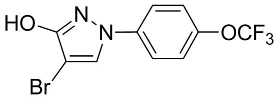

Yellow solid; Yield 71%; m.p. 198-199°C; ^{1}H NMR (400 MHz, DMSO-*d*$_6$) *δ* 11.52 (s, 1H, OH), 7.72 (s, 1H, pyrazole-H), 7.49 (d, *J* = 8.4 Hz, 2H, Ar-H), 7.34 (d, *J* = 8.4 Hz, 2H, Ar-H).

4-Bromo-1-(4-chlorophenyl)-1*H*-pyrazol-3-ol (IVd)

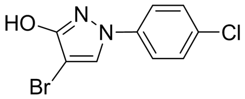

Yellow solid; Yield 78%; m.p. 192-193°C; ^{1}H NMR (400 MHz, $CDCl_3$) *δ* 7.63 (s, 1H, pyrazole-H), 7.39-7.19 (m, 4H, Ar-H).

4-Bromo-1-(3-(trifluoromethyl)phenyl)-1*H*-pyrazol-3-ol (IVe)

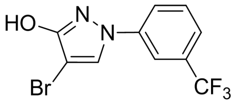

Yellow solid; Yield 72%; m.p. 195-196°C; ^{1}H NMR (400 MHz, DMSO-*d*$_6$) *δ* 9.12(s, 1H, OH), 7.74 (s, 1H, pyrazole-H), 7.65-7.19 (m, 4H, Ar-H).

4-Bromo-1-(4-fluoro-3-(trifluoromethyl)phenyl)-1*H*-pyrazol-3-ol (IVf)

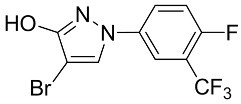

Yellow solid; Yield 70%; m.p. 201-202°C; ^{1}H NMR (400 MHz, DMSO-*d*$_6$) *δ* 10.67 (s, 1H, OH), 7.68 (s, 1H, pyrazole-H), 7.65-7.19 (m, 3H, Ar-H).

General procedure for the synthesis of intermediate 5

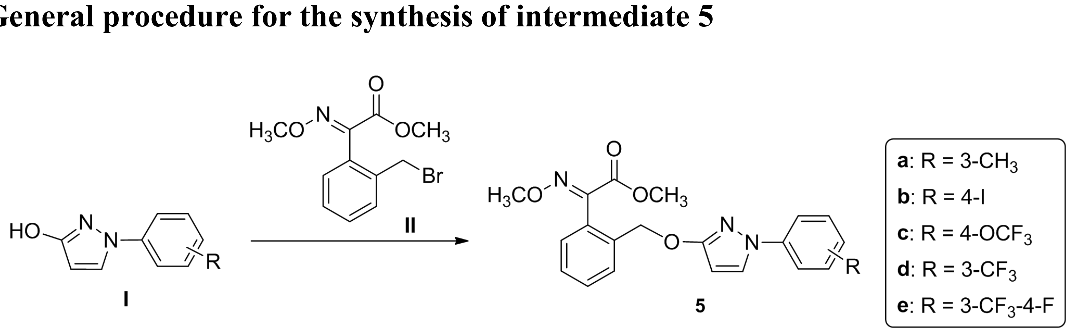

Compound **5** was synthesized according to the reported methods[2]. To a solution of **I** (1.0 mmol) in acetone (30 mL) was added K_2CO_3 (1.5 mmol). The mixture was refluxed for 15 min and **II** (1.05 mmol) was added slowly. The mixture was refluxed for 4 h, filtered, and evaporated under reduced pressure. The residue was purified by a silica-gel column chromatography (petroleum ether/EtOAc = 10:1) to afford **5**.

(*E*)-Methyl 2-(methoxyimino)-2-(2-(((1-(*m*-tolyl)-1*H*-pyrazol-3-yl)oxy)methyl)phenyl)acetate (5a)

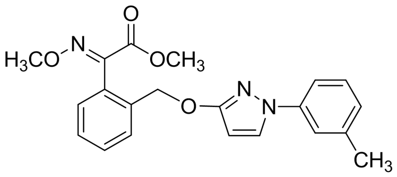

White solid; Yield 84%; m.p. 69-70°C; ^{1}H NMR (400 MHz, $CDCl_3$) δ 7.60 (d, J = 2.4 Hz, 1 H, pyrazole-H), 7.54-6.91(m, 8H, Ar-H), 5.74(d, J = 2.4 Hz, 1 H, pyrazole-H), 5.10 (s, 2H, CH_2), 3.95 (s, 3H, OCH_3), 3.74 (s, 3H, OCH_3), 2.31 (s, 3H, CH_3); ^{13}C NMR (100 MHz, $CDCl_3$) δ 164.0, 163.4, 149.5, 140.0, 135.3, 129.5, 129.2, 128.6, 128.4, 127.9, 127.8, 126.2, 118.7, 115.7, 93.8, 69.1, 63.8, 53.0, 50.7, 21.5.

(*E*)-Methyl 2-(2-(((1-(4-iodophenyl)-1*H*-pyrazol-3-yl)oxy)methyl)phenyl)-2-(methoxyimino) acetate (5b)

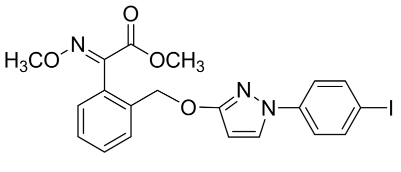

White solid; Yield 85%; m.p. 102-103°C; ^{1}H NMR (400 MHz, $CDCl_3$) δ 7.55 (d, J = 2.8 Hz, 1 H, pyrazole-H), 7.49-7.08 (m, 8H, Ar-H), 5.75 (d, J = 2.8 Hz, 1H, pyrazole-H), 5.08 (s, 2H, CH_2), 3.93 (s, 3H, OCH_3), 3.72 (s, 3H, OCH_3); ^{13}C NMR (100 MHz, $CDCl_3$) δ 163.0, 162.1, 148.2, 137.9, 133.5, 131.0, 128.4, 128.1, 127.5, 127.4, 126.8, 126.5, 118.0, 117.1, 93.5, 67.9, 62.5, 51.7.

(*E*)-Methyl 2-(methoxyimino)-2-(2-(((1-(4-(trifluoromethoxy)phenyl)-1*H*-pyrazol-3-yl)oxy) methyl)phenyl)acetate (5c)

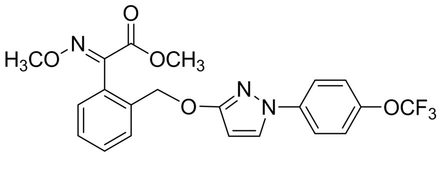

White solid; Yield 82%; m.p. 82-83°C; ^{1}H NMR (400 MHz, $CDCl_3$) δ 7.58 (d, J = 2.4 Hz, 1 H, pyrazole-H), 7.52-7.11 (m, 8H, Ar-H), 5.77 (d, J = 2.4 Hz, 1 H, pyrazole-H), 5.10 (s, 2H, CH_2), 3.95 (s, 3H, OCH_3), 3.75 (s, 3H, OCH_3); ^{13}C NMR (100 MHz, $CDCl_3$) δ 164.2, 163.4, 149.5, 146.3, 138.7, 135.2, 129.8, 129.5, 128.6, 128.4, 128.0, 127.9, 122.1, 118.8, 94.7, 69.1, 63.8, 53.0.

(*E*)-Methyl 2-(methoxyimino)-2-(2-(((1-(3-(trifluoromethyl)phenyl)-1*H*-pyrazol-3-yl)oxy) methyl)phenyl)acetate (5d)

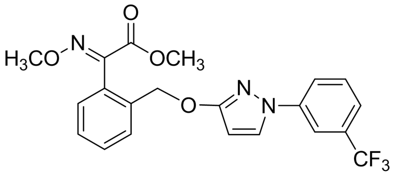

White solid; Yield 82%; m.p. 104-105°C; ^{1}H NMR (400 MHz, $CDCl_3$) δ 7.88 (d, J = 3.6 Hz, 1 H, pyrazole-H), 7.77-7.22 (m, 8H, Ar-H), 5.91 (d, J = 3.6 Hz, 1 H, pyrazole-H), 5.23 (s, 2H, CH_2), 4.07 (s, 3H, OCH_3), 3.87 (s, 3H, OCH_3); ^{13}C NMR (100 MHz, $CDCl_3$) δ 164.0, 163.0, 149.1, 140.0, 134.7, 132.1, 131.7, 131.3, 129.6, 129.5, 129.0, 128.2, 128.1, 127.7, 127.6, 125.2. 121.6, 121.3, 121.2, 120.0, 114.2, 114.1, 114.0, 94.8, 68.8, 63.4, 52.6.

(*E*)-Methyl 2-(2-(((1-(4-fluoro-3-(trifluoromethyl)phenyl)-1*H*-pyrazol-3-yl)oxy)methyl) phenyl)-2-(methoxyimino)acetate (5e)

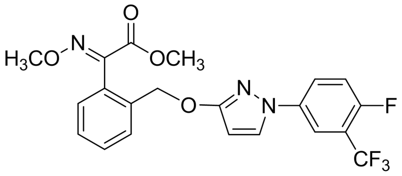

White solid; Yield 80%; m.p. 107-108°C; ^{1}H NMR (400 MHz, $CDCl_3$) δ 7.84 (d, J = 3.6 Hz, 1 H, pyrazole-H), 7.73-7.22 (m, 7H, Ar-H), 5.91 (d, J = 3.6 Hz, 1 H, pyrazole-H), 5.20 (s, 2H, CH_2), 4.06 (s, 3H, OCH_3), 3.87 (s, 3H, OCH_3); ^{13}C NMR (100 MHz, $CDCl_3$) δ 164.0, 163.0, 149.0, 136.0, 134.6, 129.5, 129.1, 128.2, 128.1, 127.6, 127.5, 122.2, 122.1, 117.6, 117.4, 116.1, 116.0, 94.8, 68.8, 63.4, 52.6.

References

1. Li, Y. H., Liu, R., Yan, Z. W., Zhang, X. N. & Zhu, H. J. Synthesis, crystal structure and fungicidal activities of new type oxazolidinone-based strobilurin analogues. *Bull. Korean Chem. Soc.* **31**, 3341-3347 (2010).
2. Liu, Y. Y. *et al.* Synthesis and fungicidal activity of novel chloro-containing

1-aryl-3-oxypyrazoles with an oximino ester or oximino amide moiety. *Molecules* **19**, 8140-8150 (2014).

X-Ray Crystallographic Analysis of 1f, 2b and 3b

Single crystals were obtained by evaporation of a solution of **1f**, **2b** and **3b** in methanol at room temperature.

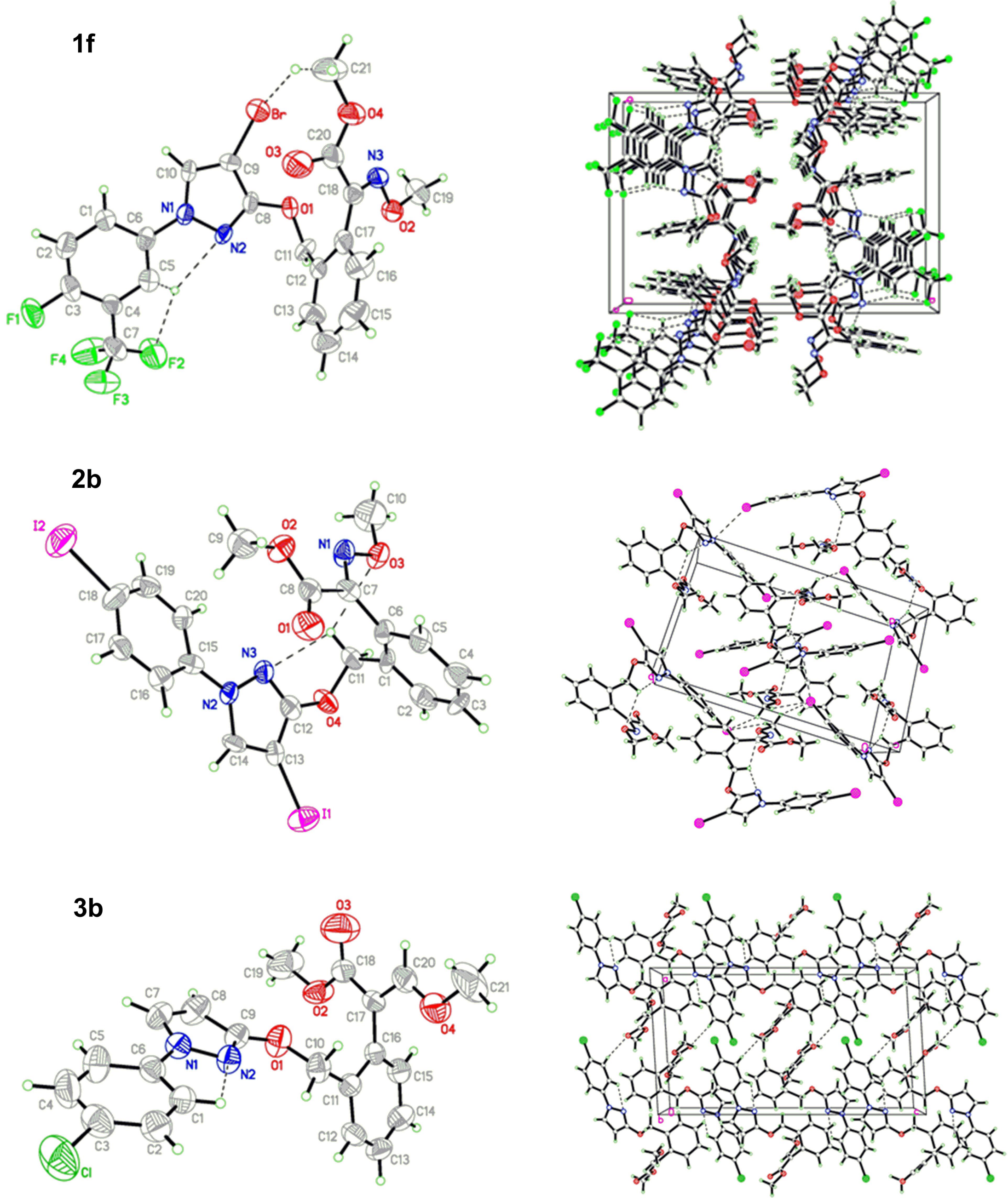

Figure 1. X-ray structures (ellipsoids are shown at the 50% probability level) and packing diagrams (hydrogen bonds are shown as dashed lines).

Table 1 Crystallographic data and structure refinement for **1f**, **2b** and **3b**

	**1f**	**2b**	**3b**
Empirical formula	$C_{21}H_{15}BrF_4N_3O_4$	$C_{20}H_{17}I_2N_3O_4$	$C_{21}H_{19}ClN_2O_4$
CCDC number	1433534	1433532	1433533
Formula weight	529.27	617.17	398.83
Temperature [K]	293(2)	293(2)	293(2)
Wavelength [Å]	0.71073	0.71073	0.71073
Crystal system	Monoclinic	Monoclinic	Monoclinic
Space group	P21/c	P21/n	P21/c
Unit cell dimensions			
a [Å]	20.446 (4)	11.639 (2)	11.773(2)
b [Å]	13.551(3)	16.736(3)	7.8560(16)
c [Å]	7.0590(14)	12.343(3)	21.480(4)
α [º]	90.00	90.00	90.00
β [º]	90.10(3)	116.01(3)	93.27(3)
γ [º]	90.00	90.00	90.00
Volume [Å^3]	1955.8(7)	2160.8(7)	1983.4(7)
Z	4	4	4
ρ_{calcd} [g cm^{-3}]	1.797	1.897	1.336
μ [mm^{-1}]	2.177	2.941	0.222
F(000)	1060	1184	832
Crystal size [mm^3]	0.20 × 0.10 × 0.10	0.20 × 0.10 × 0.10	0.30 × 0.20 × 0.10
θ range [º] for data collection	1.99 to 28.18	2.01 to 25.42	1.73 to 25.36
Index ranges	-24≤h≤0	0≤h≤14	0≤h≤14
	-16≤k≤0	0≤k≤20	0≤k≤9
	-9≤l≤9	-14≤l≤13	-25≤l≤25
Reflections collected	4077	4139	3824
Independent reflections	3967 [R_{int} = 0.0997]	3941 [R_{int} = 0.0990]	3637 [R_{int} = 0.0657]
Max. and min. transmission	0.8117/0.6699	0.7574/0.5908	0.9781/0.9364
Data/restraints/parameters	3967 /2/ 298	3941/0/262	3637/1/253
Goodness-of-fit on F^2	1.004	1.009	1.008
Final R indices [I>2σ(I)]; R_1, wR_2	0.0772, 0.1344	0.0791, 0.1683	0.0771, 0.1690
R_1, wR_2 (all data)	0.1942, 0.1657	0.1544, 0.1963	0.1452, 0.1997
Largest diff. peak and hole [e·Å^{-3}]	0.383 and -0.404	0.522 and -0.305	1.051 and -0.309

Table 2 Parameters (Å, °) for the intra- and intermolecular interactions in **1f**, **2a** and **3b**

Comp. No.	D-H···A	D-H	H···A	D···A	D-H···A	symmetry code
**1f**	C1-H1A···O3	0.9300	2.4700	3.401(8)	176.00	x,1/2-y, -1/2+z
	C5-H5A···F2	0.9300	2.3300	2.657(8)	100.00	/
	C5-H5A···N2	0.9300	2.4200	2.754(8)	101.00	/
	C10-H10A···O3	0.9300	2.4100	3.262(9)	153.00	x,1/2-y, -1/2+z
	C14-H14A···F1	0.9300	2.5000	3.402(9)	164.00	2-x,1/2+y,3/2-z
	C14-H14A···F4	0.9300	2.5400	3.225(9)	130.00	2-x,1-y,1-z
	C16-H16A···N2	0.9300	2.6200	3.338(9)	135.00	x,y,1+z
	C21-H21B···Br	0.9600	2.9300	3.676(8)	135.00	x,y,1+z
	C21-H21C···Br	0.9600	2.7900	3.484(8)	130.00	/
**2b**	C11-H11A···O3	0.9700	2.5800	3.044(16)	110.00	/
	C11-H11A···N3	0.9700	2.4400	2.782(15)	101.00	/
	C14-H14A···O1	0.9300	2.4000	3.289(16)	161.00	1-x,1-y,-z
	C16-H16A···O1	0.9600	2.5400	3.429(15)	160.00	1-x,1-y,-z
**3b**	C1-H1A···N2	0.9300	2.4500	2.779(5)	101.00	/
	C2-H2B···O3	0.9300	2.4900	3.334(6)	150.00	1+x, y, z
